# Transcriptome and network analyses in *Saccharomyces cerevisiae* reveal that amphotericin B and lactoferrin synergy disrupt metal homeostasis and stress response

**DOI:** 10.1038/srep40232

**Published:** 2017-01-12

**Authors:** Chi Nam Ignatius Pang, Yu-Wen Lai, Leona T. Campbell, Sharon C.-A. Chen, Dee A. Carter, Marc R. Wilkins

**Affiliations:** 1 School of Biotechnology and Biomolecular Sciences, The University of New South Wales, Kensington, New South Wales, Australia; 2 School of Life and Environmental Sciences, University of Sydney, Sydney, New South Wales, Australia; 3 Marie Bashir Institute for Infectious Diseases and Biosecurity, University of Sydney, Sydney, NSW, Australia; 4 Centre for Infectious Diseases and Microbiology, Institute of Clinical Pathology and Medical Research, Westmead Hospital, Sydney Medical School, University of Sydney, Westmead, NSW, Australia

## Abstract

Invasive fungal infections are difficult to treat. The few available antifungal drugs have problems with toxicity or efficacy, and resistance is increasing. To overcome these challenges, existing therapies may be enhanced by synergistic combination with another agent. Previously, we found amphotericin B (AMB) and the iron chelator, lactoferrin (LF), were synergistic against a range of different fungal pathogens. This study investigates the mechanism of AMB-LF synergy, using RNA-seq and network analyses. AMB treatment resulted in increased expression of genes involved in iron homeostasis and ATP synthesis. Unexpectedly, AMB-LF treatment did not lead to increased expression of iron and zinc homeostasis genes. However, genes involved in adaptive response to zinc deficiency and oxidative stress had decreased expression. The clustering of co-expressed genes and network analysis revealed that many iron and zinc homeostasis genes are targets of transcription factors Aft1p and Zap1p. The *aft1Δ* and *zap1Δ* mutants were hypersensitive to AMB and H_2_O_2_, suggesting they are key regulators of the drug response. Mechanistically, AMB-LF synergy could involve AMB affecting the integrity of the cell wall and membrane, permitting LF to disrupt intracellular processes. We suggest that Zap1p- and Aft1p-binding molecules could be combined with existing antifungals to serve as synergistic treatments.

Fungal infections, including invasive fungal disease, are on the rise, are often difficult to treat^1^, and there is a sparse drug development pipeline for new antifungals^2^. The amphipathic polyene amphotericin B (AMB) for many years has been the “workhorse” of antifungal therapy and considered the gold-standard treatment for most fungal infections due its broad antifungal spectrum of activity. However, it is toxic and difficult to administer, requiring intravenous infusion and continual clinical monitoring^3^. While less toxic, other antifungals are less potent, have a limited spectrum of activity or induce resistance, and there is invariably a trade-off between toxicity and efficacy. A promising strategy to enhance efficacy whilst reducing drug toxicity is to combine known antifungals with either existing drugs or small molecule adjuvants, with the aim of producing synergistic combinations^4^,^5^,^6^. Systematic screening for effective drug-drug and drug-adjuvant pairs have found synergistic interactions to be relatively uncommon (hit rates of 4–10%)^6^. While some agents, such as those that target the fungal cell membrane or cell wall have a higher tendency to participate in a synergistic combination^4^,^6^, the mechanistic basis for synergy is poorly understood^4^. Recent high-throughput chemical-genetic screens have identified genes that when deleted increase susceptibility to a single drug^7^,^8^ or combination of drugs^5^, assisting with the understanding of the mechanisms of synergy and prediction of novel synergistic drug pairs^9^,^10^. New drugs can be developed or existing drugs can be repurposed to realize the synergistic mechanism adding to therapeutic antifungal options^11^.

One approach to understand mechanisms of synergy is through ‘Omic’ analyses. These allow global changes in intracellular gene or protein abundance to be identified, enabling data-driven identification of the pathways that are disrupted by drug-drug interactions^12^. For example, transcriptome and proteome analyses have been used to investigate the mechanism of synergy between berberine and fluconazole in fluconazole-resistant *Candida albicans*^13^,^14^. Transcriptomics and proteomics have advantages over chemical-genetic screens as they require fewer resources and can be applied to different organisms or to drug-resistant strains. Known drug targets and genes or proteins that are differentially expressed following drug treatment can be mapped onto genetic, regulatory or protein-protein interaction networks to generate hypotheses on how synergy arises via the disruption of multiple interdependent pathways or processes^15^,^16^. Critically, it is known that genes and proteins involved in virulence and drug resistance can interact with each other physically or functionally within highly connected networks^17^,^18^, and that drug targets are often in close proximity in a network to genes or proteins that are differentially expressed upon drug treatment^19^. Therefore, the analysis of transcriptomic or proteomic data in the context of networks can reveal regions perturbed upon drug treatment, help characterize the mode-of-action of drugs and predict new drug targets^19^,^20^.

To identify synergistic combinations, we recently screened 30 pairs of various known antifungal agents and iron chelators^3^. AMB and lactoferrin (LF), a multifunctional iron chelating and antimicrobial protein present in milk and tears^21^, interacted synergistically against the model yeast *Saccharomyces cerevisiae* and the pathogen *Cryptococcus*. This combination has been previously shown to be synergistic against *C. albicans*^22^ and *Aspergillus fumigatus*^23^, suggesting it may have broad spectrum of activity^3^. The addition of exogenous iron rescued cells from the action of LF alone but could not rescue AMB-LF synergy, suggesting the synergistic mechanism involves additional properties of LF other than iron chelation^3^. LF alone has a relatively high MIC for *Saccharomyces* and *Cryptococcus* and this was greatly reduced by the presence of AMB, indicating that AMB potentiated activity, but the mechanism of this is unknown^3^.

*Saccharomyces cerevisiae* has rich annotation resources^24^ and the most comprehensively mapped intracellular networks of any eukaryotic organism^18^,^25^. As it shares evolutionarily conserved genes, pathways and networks with pathogenic fungi including *Cryptococcus, Candida* and *Aspergillus* where genomic resources are relatively lacking^17^, it is a useful model for analyzing cellular responses to antifungal agents. In this study, we first used RNA-seq to explore the transcriptomic response to drug synergy induced by AMB and LF. Transcriptome data indicated that AMB and LF interfered with the stress responses associated with dysregulated iron and zinc homeostasis. By network analysis the transcription factors Aft1p and Zap1p were identified as key regulators of these processes. Knockout of *AFT1* or *ZAP1* caused increased susceptibility to AMB, H_2_O_2_ and other stressing agents, suggesting that they or their homologs may be suitable targets for adjunct therapies with AMB.

## Results

### Differential gene expression induced by AMB and AMB-LF

Wild type *S. cerevisiae* cultures were treated with AMB or AMB-LF according to the Materials and Methods (and Supplementary Figure S1). AMB and AMB-LF cultures were both harvested at their respective ID_20_, where levels of cellular stress would be similar and any confounding effects due to more rapid inhibition and death in the synergistic combination would be minimized. The transcriptome of AMB treatment had 907 and 921 genes with a significantly increased, or decreased level of expression, respectively, compared to the matched control. In the AMB-LF treatment, there were 748 and 689 genes with an increased, or decreased level of expression, respectively, compared to a separate matched control (adjusted p-values < 0.05, Supplementary Figure S1–3 and Data S1). No genes were significantly differentially expressed under LF treatment (Supplementary Figure S4), which was consistent with failure of LF to suppress *S. cerevisiae* growth compared to the control (Supplementary Figure S1). This treatment was therefore not further studied, and comparisons were limited to the AMB and AMB-LF treatments.

### Gene Ontology analysis reveals enrichment of metal ion homeostasis and stress response among differentially expressed and co-regulated genes

To identify the biological processes associated with treatment using AMB alone or in combination with LF, the two lists of differentially expressed genes (with increased or decreased expression) from both the AMB only and AMB-LF treatments (see above) were separately analyzed for enrichment of Gene Ontology (GO) terms^26^. We used the ClueGO tool to search for enriched GO terms and took the most significant GO term from each group as a representative^27^ (see Supplementary Methods). GO terms with adjusted p-values of < 0.05 indicate significant enrichment in query genes compared to the background of the total set of yeast genes.

**Figure 1:**
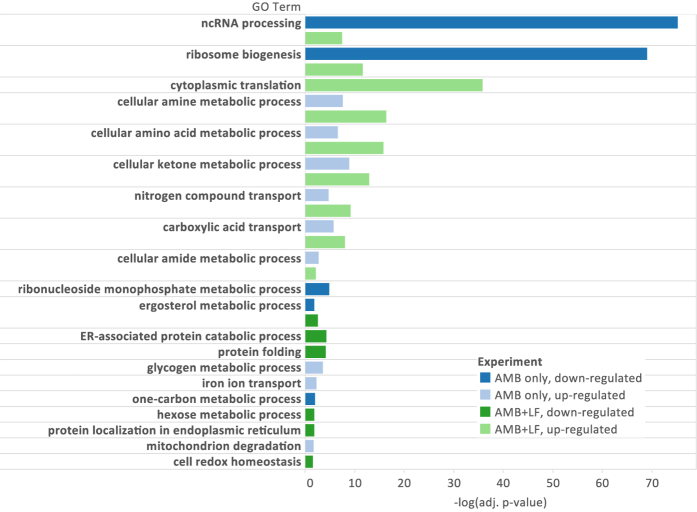
Functional enrichment of genes differentially expressed in response to AMB alone and in combination with LF. Significantly enriched representative GO terms (adjusted p-value < 0.05) are listed on the y-axis, and the negative log of the adjusted p-value (base 10) is represented by the x-axis. Where multiple GO terms represented the same group of genes, the most significant GO term was considered as representative and shown for this analysis. Blue = genes differentially expressed in response to AMB alone; green = genes differentially expressed in response to AMB-LF; light colours = increased gene expression; dark colours = decreased gene expression.

Genes with increased expression following AMB treatment were associated with nine significantly enriched biological processes, including metabolic processes (amine, amino acids, ketone, amide and glycogen), transport of iron, nitrogen compounds, and carboxylic acid and mitochondrion degradation (Fig. 1). In addition, five significantly enriched GO biological processes were identified among genes with decreased expression, including those related to ribosomal biogenesis, ncRNA processing and metabolic pathways that involve ribonucleoside monophosphate, one-carbon metabolism and ergosterol (Fig. 1).

Following AMB-LF treatment, genes with increased expression were significantly enriched for processes involved in maintaining protein expression and metabolism (Fig. 1 and Supplementary Data S1). These included ribosome biogenesis, cytoplasmic translation, ncRNA processing, metabolic pathways (amine, amino acid, ketone and amide), and transport of nitrogen compounds and anions. Genes with decreased expression were enriched for processes involved in the redox stress response and protein quality control in the endoplasmic reticulum (ER) (Fig. 1). These included cell redox homeostasis, protein folding and catabolism in the ER, and other metabolic processes that involve phospholipids, ergosterol and hexose.

**Figure 2:**
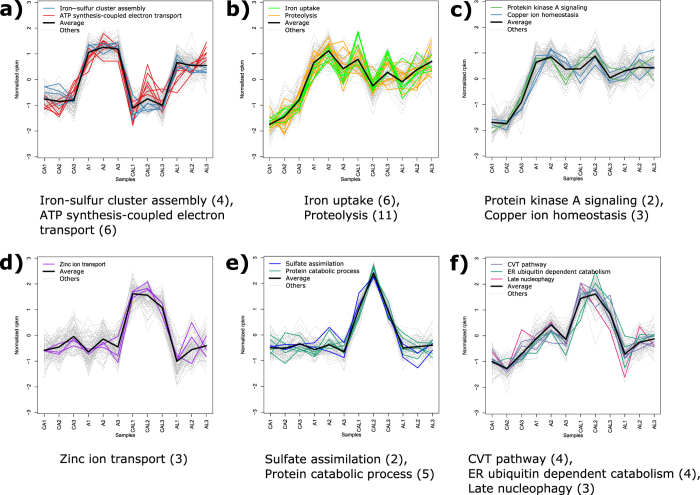
Clusters of co-expressed genes from self-organizing maps enriched for metal ion homeostasis and stress response. Clusters from self-organizing maps, produced using differentially expressed transcripts from the AMB treatment (panels a to c) and AMB-LF treatment (panels d to f), show enrichment for specific biological processes. The y-axis represents the scaled Reads Per Kilobase per Million mapped reads (rpkm), the x-axis represents the four treatment types with the biological triplicates numbered; CA = matched control for AMB, A = AMB, CAL = matched control for AMB-LF, AL = AMB-LF. Black lines represent the average gene expression; coloured lines are genes associated with enriched biological processes labeled in the legends and grey lines represent other genes. The enriched biological process associated with each cluster and the number of genes associated with each process (in brackets) is shown below each panel. The genes associated with each cluster are shown in Table 1, and the complete SOMs are in Supplementary Figure S5.

To assess how co-regulated processes contributed to the response to drug treatments, self-organizing maps (SOMs) were used to cluster genes based on their profile of expression across all samples, with the assumption that genes involved in similar processes or pathways are likely to be co-regulated via a shared mechanism. Transcript data from the AMB and AMB-LF treatments were first organized in two SOMs to identify clusters of genes with correlated expression patterns (Supplementary Figure S5 and Data S2, S3). Each cluster from each map was then analyzed for enriched GO biological processes. Since changes in biological processes can represent the effect of the drug or the disruption of processes downstream, such as inhibition of cell growth, we focused on a small set of processes likely to be a direct response to AMB^28^ and LF^29^,^30^. These included metal ion homeostasis, ER-associated ubiquitin-dependent protein catabolic process, protein refolding, and cellular response to redox homeostasis (Table 1, Fig. 2 and Supplementary Data S3). Effects that were likely to be secondary or downstream as a result of drug treatment, such as changes in ncRNA processing, ribosome biogenesis, translation and the metabolism of nitrogen and amino acids, are not further discussed but are included as Supplementary Data S3.

### AMB treatment activates expression of metal ion homeostasis genes, which becomes dysregulated with AMB-LF treatment

Cells subjected to AMB treatment had an increased, coordinated expression of genes involved in metal ion homeostasis (Table 1). In contrast, the mRNAs associated with many of these processes were down-regulated or were no longer differentially expressed during AMB-LF treatment. Biogenesis of iron-sulfur clusters in the mitochondria is required for the activation of iron import genes^31^. Consistent with this, the expression of genes involved in iron-sulfur cluster assembly, including those in the mitochondria, was up-regulated in both the AMB- (Fig. 2a) and the AMB-LF-treated cells (Supplementary Data S3). While AMB treatment increased the expression of genes involved in iron uptake and transport of siderophores (Fig. 2b), which are small molecules that bind to iron^32^, these genes had little or no differential expression upon treatment with AMB-LF. The expression of genes involved in copper ion transport was only up-regulated with AMB treatment but not differentially expressed under AMB-LF treatment (Fig. 2c). The expression of zinc transport genes was only down-regulated by AMB-LF treatment (Fig. 2d).

### Adaptive responses to zinc-deficiency are down-regulated during AMB-LF treatment

The sulfate assimilation pathway is normally repressed to limit oxidative stress during zinc deficiency^32^. Genes in this pathway were found to decrease in expression following AMB-LF treatment (Fig. 2e). As part of the unfolded protein response, genes associated with protein degradation in the ER and vacuole are normally up-regulated by zinc depletion and increased oxidative stress^32^,^33^, however genes involved in these processes were also found to be decreased in expression following AMB-LF treatment. Similarly, although genes involved in ER-associated protein catabolic process increased in expression following AMB treatment (Fig. 2a), they decreased relative to their control following AMB-LF treatment (Fig. 2e and f). Genes involved in protein degradation in the vacuole, including the cytoplasm-to-vacuole targeting (CVT) and late nucleophagy processes, also had decreased expression with AMB-LF treatment (Fig. 2f). Overall, this suggests AMB-LF treatment resulted in stress adaptation pathways that are normally coordinated with zinc deficiency to become dysregulated.

### Protein kinase A signaling and apoptosis genes are up-regulated by AMB treatment but not by AMB-LF treatment

Protein kinase A (PKA) signaling regulates stress responses. It negatively regulates genes involved in iron uptake^34^, activates production of ROS in the mitochondria and activates apoptosis when the cell is exposed to antifungal drugs^28^. Genes involved in PKA signaling and ATP synthesis-coupled electron transport in the mitochondria had increased expression following AMB treatment (Fig. 2c). Interestingly, reductive iron transport and trehalose and glycogen biosynthesis were also among the genes with increased expression under AMB treatment (Supplementary Data S2 and S3); these pathways are known to be repressed by PKA^34^. However, PKA transcripts were no longer differentially expressed following AMB-LF treatment. Since PKA signaling is responsible for activating apoptosis via actin aggregation and the accumulation of ROS in the mitochondria, we analyzed the genes known to be involved with these apoptosis and oxidative stress responses^24^,^28^. Although 15 genes involved in apoptosis were up-regulated by AMB treatment, following AMB-LF treatment most apoptotic genes were not differentially expressed (Supplementary Table S2). We also observed a lack of an acute response to oxidative stress under both AMB and AMB-LF treatments (Supplementary Table S3).

### Network analysis identifies Aft1p and Zap1p as regulators of gene expression in response to AMB and AMB-LF treatments

We hypothesized that transcription factors might be responsible for regulating the co-expression of genes in particular SOM clusters, and if so the target genes of a transcription factor would be over-represented in that cluster. Analysis of all the clusters identified 12 transcription factors whose targets were enriched among the co-expressed genes within their cluster (Supplementary Table S4). Of these, we focused on the transcription factors Aft1p and Zap1p since they are known to regulate the expression of genes involved in the enriched GO terms iron uptake and zinc ion transport^32^, respectively (Table 1). Although this analysis identified Aft2p, we focused the analysis on Aft1p as these are paralogs and share a high number of gene targets^32^ and only *AFT1* was significantly differentially expressed.

**Figure 3:**
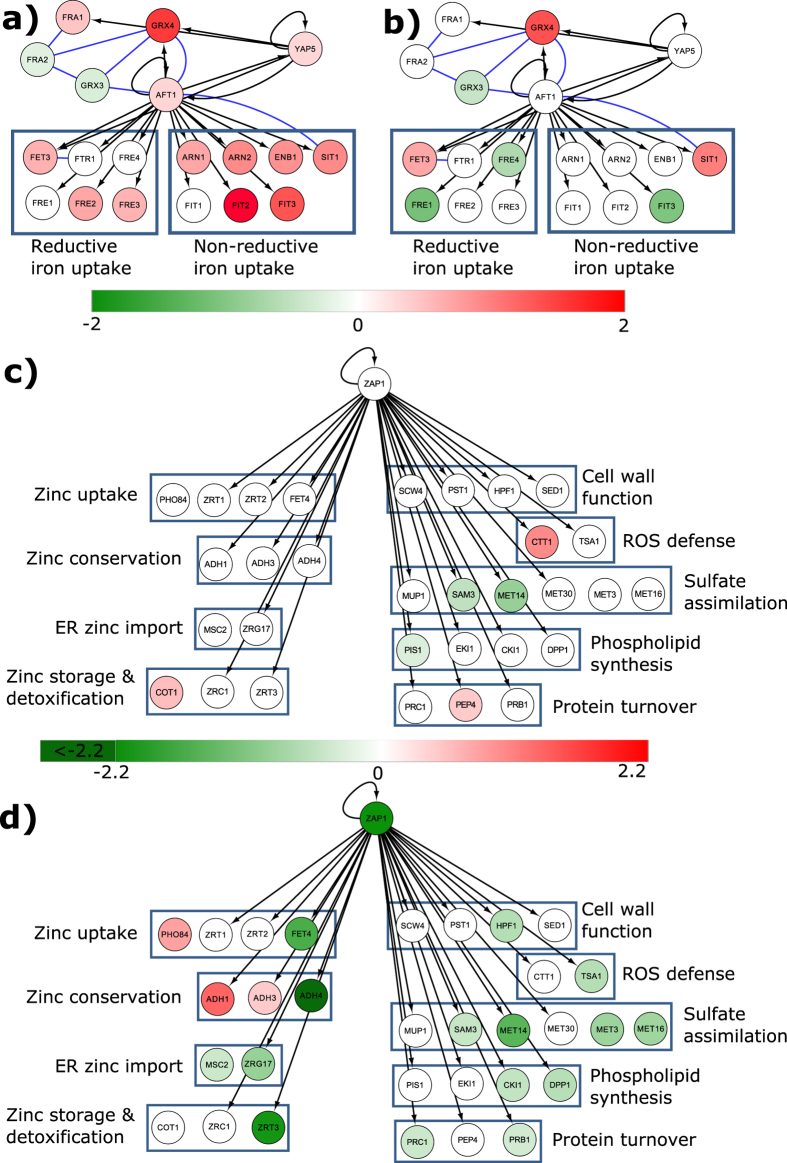
The transcriptional regulatory network of Aft1p and Zap1p and the expression of their target genes indicate dysregulation of metal ion homeostasis during synergistic AMB-LF treatment. The Yeastract transcriptional regulatory network database was used to identify the genes targeted by transcription factors Aft1p and Zap1p under AMB and AMB-LF treatments. Differential expression of *AFT1* and target genes under (**a**) AMB and (**b**) AMB-LF treatment. Iron uptake genes are activated by Aft1p during AMB treatment, but iron uptake is comparatively down-regulated during AMB-LF treatment. Differential expression of Zap1p and target genes following (**c**) AMB and (**d**) AMB-LF treatments. *ZAP1* and 16 out of 32 Zap1p-targeted genes decreased in expression under AMB-LF treatment. In the network, each circle represents a gene with the gene name labeled. Bars indicate log_2_ fold-change for each network; green = negative fold-change, red = positive fold-change and white = no or statistically insignificant fold-change. Blue lines indicate protein-protein interactions. Black arrows show transcription factor regulation, with the arrow pointing towards the target gene.

Network visualization was used to further understand how the expression of *AFT1*, and the Aft1p-binding targets involved in iron homeostasis, was affected in the two treatments (Fig. 3). During AMB treatment there was increased expression of *AFT1*, along with 9 of its 13 target genes involved in iron uptake. There was also increased expression of *FRA1* and *GRX4* (Fig. 3a). This was accompanied by increased expression of *YAP5*, which encodes the transcription factor that activates *GRX4* expression^35^. Grx4p and Fra1p interact to form a complex that facilitates the export of Aft1p from the nucleus into the cytoplasm during iron-replete conditions^32^,^36^. Together, these results suggest that during AMB treatment there is dysregulation of iron homeostasis in that Aft1p is activating the expression of iron uptake genes in the nucleus but *FRA1* and *GRX4* may be counteracting this by the export of Aft1p. In contrast, and consistent with the GO term analysis results outlined above, AMB-LF treatment led to *AFT1* and 11 of the 13 iron uptake genes being either not differentially expressed or having decreased expression (Fig. 3b).

The network for Zap1p highlighted a different pattern of regulation compared to Aft1p. Zap1p directly senses zinc levels and is the major transcription factor that regulates zinc uptake and pathways involved in adaptation to the various stresses caused by zinc deficiency, such as oxidative stress tolerance and sulfate assimilation^32^. Under AMB treatment, *ZAP1* was not differentially expressed and the majority of Zap1p target genes involved in zinc homeostasis had no significant change in expression (Fig. 3c). During AMB-LF treatment, the expression of *ZAP1* and 16 out of 32 genes involved in the adaptation to zinc deficiency decreased (Fig. 3d). These include genes involved in zinc transport and conservation, sulfate metabolism, ROS defense, phospholipid synthesis, protein turnover and cell-wall functions^32^. The only genes to increase in expression were *ADH1, ADH3* and *PHO84. ADH1* and *ADH3* encode zinc binding enzymes and are repressed by Zap1p^32^, therefore their increased expression is consistent with the decreased expression of *ZAP1. PHO84* encodes a low-affinity zinc and phosphate transporter but its expression is not regulated by intracellular levels of zinc and is therefore unlikely to be regulated by Zap1p^32^.

### Zinc and iron homeostasis are critical processes in AMB-LF synergy

**Figure 4:**
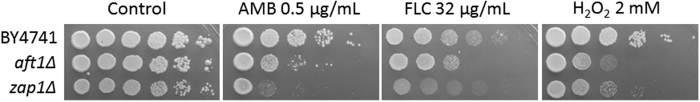
a*ft1Δ* and *zap1Δ* mutants have increased susceptibility to AMB, FLC and H_2_O_2_. Growth of *aft1Δ* and *zap1Δ* strains compared to the *S. cerevisiae* haploid wild type (BY4741) background. Serial 10-fold dilutions of the strains are plated from left (1 × 10^6^ cells/mL in 5  μL spot) to right on synthetic complete agar with AMB (0.5 μg/mL), FLC (32 μg/mL) or H_2_O_2_ (2 mM). Growth at 30 °C was recorded at 3 days (control, AMB, FLC) or 2 days for H_2_O_2_ treatment.

Since iron and zinc homeostasis were dysregulated by the synergistic AMB-LF combination, we hypothesized that knocking out *AFT1* and *ZAP1* could recapitulate drug synergy with AMB. We therefore tested the sensitivity of *aft1Δ* and *zap1Δ* gene knockout strains to AMB (Fig. 4 and Supplementary Figure S6). The *zap1Δ* mutant was severely retarded in growth compared to control strain BY4741, and the *aft1Δ* mutant had reduced growth but this was less severe. The relevance of the plate-based assays was confirmed by growing *aft1Δ, zap1Δ* and wild type control in the presence of AMB-LF (Supplementary Figure S6). As expected, the growth of knockout strains was retarded by the synergistic combination. Together, this confirms that the regulation of iron and zinc homeostasis by Aft1p and Zap1p is important for the cellular response to AMB, with zinc homeostasis emerging as vital for AMB tolerance. The *aft1Δ* and *zap1Δ* strains also had increased susceptibility to oxidative stress (H_2_O_2_) and fluconazole (FLC). The latter is possibly due to increased ergosterol depletion, as several of the enzymes involved in ergosterol biosynthesis require heme or iron cofactors to function^37^. The *aft1Δ* strain had increased susceptibility to many other stressors, including caffeine, calcofluor white, NaCl, NaNO_2_ and SDS, while the *zap1Δ* strain was also susceptible to caffeine, calcofluor white and 37 °C (Supplementary Figure S6). These results suggest Aft1p and Zap1p are important for stress response in general and could be targets for synergistic antifungal therapy in combination with AMB.

To finally confirm the involvement of Aft1p and Zap1p in drug synergy, we performed the same analysis as above on 9 other knockout strains (Supplementary Tables S5, S6 and Figure S6–7). We examined deletions of single genes associated with metal ion homeostasis, adaptation to metal ions deficiency and stress response (*atg1Δ, cch1Δ, met32Δ, mtd1Δ, tpk2Δ, yap5Δ, yct1Δ, yor387cΔ* and *vel1Δ*) and a double gene knockout of the paralogous pair *vel1Δ/yor387cΔ*. None of these mutant strains had increased susceptibility to AMB or other tested stresses (Supplementary Figure S6), confirming that Aft1p and Zap1p are critical for survival to drug synergy. It also suggests that the targeting of high-level regulators, such as transcription factors, is likely to be important for achieving drug synergy with AMB.

## Discussion

**Figure 5:**
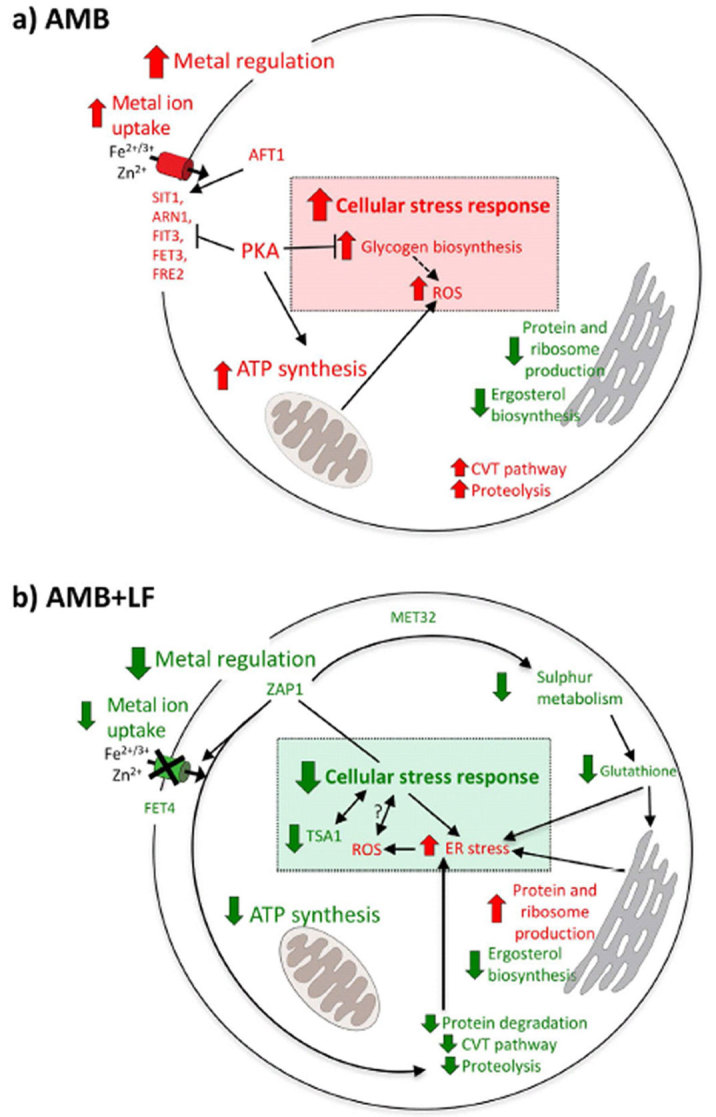
A model of the cellular response to AMB-LF synergy. (**a**) AMB alone induces a range of cellular stress responses, including increased expression of genes involved in the import of metal ions and the oxidative stress response. (**b**) During AMB-LF treatment there is decreased expression of genes involved in the uptake of iron and zinc mediated by Aft1p and Zap1p, and an overall decrease in the expression of genes involved in stress response and proteolysis. While this should lead to an accumulation of intracellular ROS and increased oxidative stress, the transcriptome indicates that the cellular stress response decreases. These results suggest general dysregulation of metal ion homeostasis and the appropriate cellular stress responses. Arrows show the direction of regulation. Arrow with blunt end = inhibitory signal; arrow with dotted line = inactivated process. Red = significant increase in the expression of gene or process; green = significant decrease in expression; black = no change in expression.

In this study, we investigated the mechanistic basis of AMB-LF drug synergy in *S. cerevisiae* through RNA-seq and network analysis. We identified a number of differentially expressed genes from each of the AMB and AMB-LF treatments and analyzed each set of genes through clustering of co-expressed genes and functional enrichment. A summary of the results is presented in Fig. 5. During AMB treatment, we observed increased expression of genes involved in iron homeostasis and apoptosis, including the CVT pathway, PKA signaling and ATP synthesis-coupled electron transport (Fig. 5a). Our results are consistent with previous microarray analyses of *S. cerevisiae* and *C. albicans* treated with AMB^38^,^39^ and the analysis of an AMB-resistant strain of *C. albicans*^40^ which showed increased expression of iron uptake genes. LF treatment produced no changes in gene expression compared to the control (Supplementary Data S1). In contrast, AMB-LF treatment resulted in a significant decrease in the expression of genes associated with zinc homeostasis and adaptation to zinc deficiency, while genes involved in iron uptake, PKA signaling and ATP synthesis no longer showed increased expression (Fig. 5b). We used network analysis to search for known transcription factor targets among each cluster of co-expressed genes. This revealed that the clusters involved in iron and zinc homeostasis contained targets of transcription factors Aft1p and Zap1p, respectively^32^. We subsequently showed the *aft1Δ* and *zap1Δ* mutant strains had increased susceptibility to AMB and H_2_O_2_ (Fig. 4), suggesting the two transcription factors have significant roles in the adaptive response to AMB treatment and in oxidative stress^32^,^41^.

LF is a strong chelator of iron and can bind other metal ions^21^. We were therefore interested in how the general regulation of metals was affected in AMB-LF synergy. During AMB-LF treatment, we observed a lack of differential expression of *AFT1* and decreased expression of *ZAP1*. This was despite Aft1p and Zap1p being required for growth during AMB treatment, as demonstrated by our analysis of *aft1Δ* and *zap1Δ* mutants (Fig. 4). This suggests that one effect of AMB-LF synergy could be the inability to sense and respond to intracellular levels of iron and zinc. AMB is known to damage the integrity of the cell membrane^42^ and cell wall^43^, and may thus facilitate the entry of LF into the cell. Once there, LF could achieve synergy by disrupting intracellular targets that normally control intracellular iron and zinc levels in response to AMB stress, as shown by the lack of differential expression of *AFT1* and the repression of *ZAP1*. This hypothesis is supported by the observation that LF alone did not affect gene expression and had little or no antifungal activity under the conditions tested, since in the absence of AMB it would be outside the cell and unable to act on intracellular targets. Further work is required to determine the movement and location of LF in the presence and absence of AMB.

There are many lines of evidence to suggest that oxidative stress is associated with AMB-LF synergistic growth inhibition. AMB alone triggers a multifaceted response (Fig. 5a). It activates stress response pathways to tolerate drug toxicity^28^, including an oxidative stress response, similar to the environmental stress response^44^. LF alone, in *C. albicans*, is known to cause oxidative stress, apoptosis and leakage of cations from the plasma membrane^30^. When AMB is combined with LF, we observed that multiple oxidative stress response pathways had decreased gene expression which likely led to decreased survival against oxidative stress. Down-regulated pathways contained genes associated with sulfate assimilation and *TSA1;* genes which are regulated by Zap1p (Fig. 5b)^32^. A less active sulfate assimilation pathway would reduce the production of glutathione (Fig. 5b), an electron donor involved in ROS detoxification and protection against oxidative stress in the ER^32^. Decreased Tsa1p is also likely to be detrimental under oxidative stress, as Tsa1p detoxifies ROS and prevents the formation of aggregated proteins which themselves can induce oxidative stress^45^ (Fig. 5b). The decreased expression of genes involved in proteolysis and protein degradation would lead to increased ER stress and the accumulation of ROS (Fig. 5b)^46^.

An important question is whether transcription factors Aft1p and Zap1p could be targets of synergistic antifungal drugs in other fungal species, since their disruption increased the sensitivity of *S. cerevisiae* to AMB. Aft1p has an ortholog in *Candida glabrata* that could serve as a synergistic target in this pathogen (Table S6), and while an Aft1p ortholog is not present in *C. albicans* the transcription factor Iro1p, which regulates iron homeostasis, is a potential synergistic drug target^47^. *ZAP1* has direct orthologs in several fungal pathogens including *C. neoformans* H99 (*ZAP104*), *C. albicans (CSR1*) and *C. glabrata (CAGL0J05060g*)^48^,^49^ (Table S6). Indeed *Zap104Δ* in *C. neoformans* was shown to have increased sensitivity to AMB compared to wild type controls^48^, replicating the phenotype seen in *S. cerevisiae*. Zap1p and Zap104p have the potential to bind small molecules and DrugBank^50^, a curated database of drugs and their molecular targets, indicates that they contain putatively druggable domains. Specifically, a C2H2 zinc finger domain present in both Zap1p and Zap104p has the potential to bind picolinic acid^51^,^52^, which is a zinc chelating agent proposed to treat acne vulgaris^52^. Therefore, Zap1p and its orthologs serve as interesting putative targets for the development of novel small molecules, which could work in combination with AMB or other existing antifungals to serve as a broad-spectrum synergistic treatment.

Our results illustrate the power of analyzing gene expression and regulatory networks to understand complex cellular processes like drug synergy. Clustering of co-expressed genes by SOMs consistently led to an increase in the identification of enriched GO terms compared to not using clustering prior to enrichment analysis (Supplementary Figure S7 and Data S4). The clustering of co-expressed genes thus provided a more powerful means to identify dysregulated stress response pathways, and identified pathways that were not seen when the analysis was restricted to differential gene expression^53^. Clustering also helped to identify Aft1p and Zap1p as important regulators of LF-mediated drug synergy. Our results are consistent with Hudson *et al*.^53^, who suggested that co-expression patterns of genes could be analyzed in the context of gene regulatory networks to identify the major effector molecules that regulate the response to treatments.

In conclusion, we have shown that AMB-LF drug synergy involves dysregulation of iron and zinc homeostasis and disruption of the oxidative stress response. The expression of genes in these stress response pathways is regulated by the transcription factors Aft1p and Zap1p, whose deletion led to increased sensitivity to AMB and oxidative stress. Novel small molecule drugs that target Aft1p or Zap1p or their orthologs could be studied in combination with existing antifungals to develop new synergistic antifungal treatments.

## Materials and Methods

### Strains, culture, and agents

*S. cerevisiae* S288C strain was used for all RNA-seq analyses. Knockout mutants in the MATa BY4741 strain background were obtained from the Yeast Deletion Project^54^. Cells were grown in RPMI-1640 at 30 °C in a shaking incubator at 180 rpm. For all assays, Amphotericin B (AMB: Sigma-Aldrich, USA) was made to a stock solution of 1,600 μg/mL. For the synergy studies and time-kill curve assays, bovine lactoferrin (LF: MP Biomedical, USA) was made to 5,120 μg/mL stock in sterile MilliQ water. Further details for this are provided in the Supplementary Materials and Methods.

### Synergy studies

To analyze the effect of synergistic drug treatment, *S. cerevisiae* S288C cells grown as above were treated with i) AMB only; ii) a growth control for (i) with no AMB treatment; iii) a synergistic combination of AMB-LF; and iv) a growth control for (iii) with no AMB-LF treatment; v) LF only; and vi) a growth control for (v) with no LF treatment. The fractional inhibitory concentrations (FIC) of AMB and LF, determined by Lai *et al*.^3^ to be the most synergistic against *S. cerevisiae*, were used in all experiments. These concentrations were 0.03 μg/mL and 2 μg/mL, respectively (Supplementary Table S1).

### Time-kill curve assay

ID_20_ (inhibition of cell growth by 20%) was chosen as the time point for RNA isolation from all treatments, as at this point cells are stressed but there should be few confounding effects of cell death, allowing a direct comparison among treatments. To determine the ID_20_, growth curves for AMB- and AMB-LF-treated cultures were established for each treatment (i to iv) by sampling 500 μL of the drug-treated and corresponding untreated cultures every 15 min following drug inoculation and performing serial 10-fold dilutions from 10^−1^ to 10^−6^ in Milli Q water. Aliquots of 100 μL were plated on SDA and colonies on plates with 30–300 cfu were counted following 48 hr of incubation at 30 °C. The resulting growth curves are shown in Supplementary Figure S1. This established an ID_20_ for AMB of 60 minutes, and AMB-LF of 50 minutes. The same incubation times were applied to each of the matched control samples. LF at the FIC did not produce an observable decrease in cell growth, and cells from the LF treatment and its matched control were grown for 50 minutes to enable comparisons with the AMB-LF treatment.

### RNA-seq library preparation and sequencing

Approximately 200 μL of freeze-dried cells were beaten using 0.5 mm glass beads and RNA was isolated with the Qiagen total RNA mini isolation kit (Qiagen, Germany). RNA-Seq was performed by the Ramaciotti Centre for Genomics using the Illumina HiSeq 2000 to generate 100 bp paired-end reads. Full details are given as Supplementary Materials and Methods. All resulting RNA-seq data have been made available in GEO, with accession number GSE80357.

### Processing of RNA-seq data

RNA-Seq data were analyzed as per Twine *et al*.^55^. All details of the analysis are provided in the Supplementary Methods. Briefly, differential gene expression and data analyses were performed using R (version 3.2.5) with EdgeR (version 3.12.0)^56^, with a threshold of adjusted p-value < 0.05 for differentially expressed genes. The R scripts used for analyzing the gene expression data are available in the Bitbucket source code repository: https://bitbucket.org/IgnatiusPang/drug_synergy.

### Gene Ontology Enrichment Analysis

For the AMB and AMB-LF treatments, genes with up- or down-regulated expression were independently analyzed for enriched Gene Ontology (GO) terms using the ClueGO^27^ tool, with the parameter kappa < 0.13. Co-expressed genes from each SOM cluster were also analyzed for enriched GO terms (adjusted p-value < 0.01). The R scripts for analyzing the GO terms enrichment are provided in the following Bitbucket source code repository: https://bitbucket.org/IgnatiusPang/drug_synergy.

### Self-Organizing Maps

Self-organizing map (SOM) analysis was used to identify clusters of genes with similar expression profiles across the different treatments. The read counts were normalized by library size and gene length, log-transformed (base 2) and scaled to a mean of 0 and standard deviation of 1 per gene. The SOM was generated using the R ‘kohonen’ library (version 2.0.19)^57^.

### Network-based Enrichment of Transcription Factor Targets and Network Visualization

Each SOM cluster was analyzed, for any of the 207 known transcription factors, for network-based enrichment of transcription factor-target gene interactions in the Yeastract^58^ and YeastMine databases^59^. For each SOM cluster and transcription factor query pair, a contingency table was tabulated by comparing the list of genes in the SOM cluster against the list of target genes for transcription factor, with the total set of yeast genes as the background list. Fisher’s exact test was used to evaluate the statistical significance based on the values in the contingency table. Significant hits that were shared between the analyses of two transcription factor-target gene databases, Yeastract^58^ (version 2013-09-27; adjusted p-value of < 0.0001) and YeastMine^59^ (adjusted p-value of < 0.05) were reported. An integrated network was constructed to visualize transcription factor-target interactions and understand any relationships with protein-protein interactions. This network was formed from a union of the Yeastract^58^ transcriptional regulatory network and the protein interaction network from Pang *et al*.^60^. It was then visualized using Cytoscape version 3.2.1^61^.

### Spot plate assays

The *aft1Δ* and *zap1Δ* single gene deletion mutants were obtained from the *Saccharomyces* genome deletion project^62^. Four primer combinations were used to verify that the gene of interest was replaced with the KanMX cassette (Table S5). Overnight broth cultures of knockout mutants and BY4741 were grown in synthetic complete (SC) medium at 30 °C, 180 rpm. Cell concentrations were standardized to 1 × 10^6^ cells/mL. Aliquots of 5 μL were serially diluted 10-fold from 10^−6^ to 10 cells/mL and spotted onto SC plates supplemented with different stress inducing agents. All plates were incubated at 30 °C for 3 days unless otherwise stated. Lactoferrin for this assay was a gift from Fonterra (Victoria, Australia) and was made to 5,120 μg/mL stock in sterile MilliQ water.

### Statistical Analyses

Unless otherwise stated, the Benjamini-Hochberg procedure was used to correct the false discovery rate for all analyses. Results were deemed statistically significant with a threshold of adjusted p-value < 0.05.

## Additional Information

Accession codes: The data discussed in this publication have been deposited in NCBI’s Gene Expression Omnibus and are accessible through GEO Series accession number GSE80357 (http://www.ncbi.nlm.nih.gov/geo/query/acc.cgi?acc=GSE80357).

**How to cite this article**: Pang, C. N. I. *et al*. Transcriptome and network analyses in *Saccharomyces cerevisiae* reveal that amphotericin B and lactoferrin synergy disrupt metal homeostasis and stress response. *Sci. Rep.* **7**, 40232; doi: 10.1038/srep40232 (2017).

**Publisher's note:** Springer Nature remains neutral with regard to jurisdictional claims in published maps and institutional affiliations.

## Supplementary Material

Supplementary Information

Supplementary Dataset 1

Supplementary Dataset 2

Supplementary Dataset 3

Supplementary Dataset 4

## Figures and Tables

**Table 1:** Genes associated with metal ion homeostasis and stress response found in self-organizing map clusters.

Figure	GO Term	GO ID	Gene Names
2a	ATP synthesis-coupled electron transport	GO:0042773	*CYT1, QCR2, QCR6, RIP1, SDH1*
2a	Iron-sulfur cluster assembly	GO:0016226	*CIA2, ISA1, ISU1, NAR1*
2b	Iron uptake	GO:0055072, GO:0015891	*ARN1, ARN2, FIT2, FIT3, FRE2, FRE3*
2b	Proteolysis involved in cellular protein catabolic process	GO:0051603	*ASI2, CUE5, HUL5, NAS2, UBP2, UBP11, UBX5, UBP15, VID28, VID30, YPF1*
2c	Copper ion homeostasis	GO:0006878	*CTR2, PIC2, SCO1*
2c	Protein kinase A signaling	GO:0010737	*TPK1, TPK2*
2d	Zinc ion transport	GO:0006829	*FET4, MSC2, ZRT3*
2e	ER-associated ubiquitin-dependent protein catabolic process	GO:0030433	*DER1, DFM1, JEM1, LCL2, UBC7*
2e	Sulfate assimilation	GO:0019379	*MET3, MET16*
2f	Late nucleophagy	GO:0044805	*ATG23, ATG31, ATG9*
2f	ER ubiquitin dependent catabolism	GO:0030433	*ADD37, ATG19, HRD1, HRD3*
2f	CVT pathway	GO:0032258	*ATG19, ATG23, ATG9, SNX4*
