# Supplementary material for: Transcriptome and network analyses in Saccharomyces cerevisiae reveal that amphotericin B and lactoferrin synergy disrupt metal homeostasis and stress response
Source: Sci Rep. 2017 Jan 12;7:40232. doi: 10.1038/srep40232 (PMC5228129; doi:10.1038/srep40232)
Supplement: Supplementary Information [file srep40232-s1.pdf]

Transcriptome and network analyses in *Saccharomyces cerevisiae* reveal that amphotericin B and lactoferrin synergy disrupts metal homeostasis and stress response

Chi Nam Ignatius Pang,[1*] Yu-Wen Lai,[2*] Leona T. Campbell,[2] Sharon C.-A. Chen,[3,4] Dee A. Carter,[2†] Marc R. Wilkins[1†]

Supplementary Information

Supplementary Materials and Methods

RNA-seq library preparation and sequencing

Approximately 200 µL of freeze-dried cells were transferred to a cryogenic storage tube and mechanically broken with ~ 200 – 250 µL RNAse Zap-treated 0.5 mm glass beads using a Minilys tissue homogeniser at 5,000 rpm. The cells were beaten for a total of 2 min and cooled on ice after every 1 min of beating. Total RNA was then isolated with the Qiagen total RNA mini isolation kit. RNA quality was analysed with a Nanodrop (Thermo Scientific) to ensure an $OD_{260/230}$ and $OD_{260/280}$ ratio between 1.8 – 2, and with a Bioanalyser (Bio-Rad) to ensure an RNA Quality Indicator (RQI) of at least 8. The concentration was checked using the Qubit BR RNA assay (InVitrogen). At least 5 µg of total RNA in a minimum concentration of 200 µg/mL was sent to the Ramaciotti Centre, UNSW, Australia, for Illumina RNA sequencing.

For RNA-Seq sample preparation of the AMB treatment and the AMB-LF treatment and their respective controls, a TruSeq RNA Library prep kit v2 (Illumina) with a poly-A pulldown was used according to the

manufacturer's instructions, with 2 µg of total RNA for each of the three biological triplicate samples as input. The libraries were enriched using 15 cycles of PCR and the insert size ranged from 80 to 330 base pair (bp). The libraries were randomized and sequenced in two separate lanes using HiSeq 2000 with the TruSeq v3 SBS reagents to generate 100 bp paired-end reads. The data were demultiplexed using Casava 1.8.2.

For LF treatment and the corresponding matching controls, a TruSeq Stranded mRNA-seq prep kit was used to prepare the RNA samples as per protocol using 1 µg of total RNA for each of the three biological triplicate samples as input. The libraries were enriched using 12 cycles of PCR to minimize the number of PCR duplicates. The libraries were multiplexed and sequenced on the same flow cell using NextSeq500 v2 reagents to generate 75 bp single reads. The data were demultiplexed using Casava 1.8.2.

Processing of RNA-seq data

RNA-seq data were analysed as per Twine *et al.* (2013)[1]. Briefly, sequencing reads for all samples were assessed for quality using FastQC (version 0.10.1; www.bioinformatics.babraham.ac.uk/projects/fastqc). All samples had an average Phred score of 30 or greater. The SolexaQA (version 2.2) was used to trim the paired-end reads using p-value < 0.05 as a cutoff and selecting high quality paired-end reads with length of at least 25 nucleotides[2]. A median of 93.26% reads across all samples were high quality paired-end reads. Filtered reads were mapped against the *S. cerevisiae* genome reference (R64-1-1 build)[3] using Bowtie (version 2-2.0.0-beta7)[4] and TopHat (version 2.0.4)[5] with options "-N 3 -library-type fr-unstranded". Aligned reads with 3 or more mismatches were discarded, and reads were aligned from the left end of the fragment to the transcript strand and right end of the fragment to the opposite strand. All high-quality paired-end reads were mapped to the *S. cerevisiae* genome. The number of reads

mapped to each gene was counted using HTSeq (version 0.5.3p9) using the options "-s no -m union -t CDS -i gene_id". Reads were mapped to the coding sequence (CDS) irrespective of the strand and reads found in ambiguous overlapping CDS were not counted. The GFF gene co-ordinates file was obtained from the *Saccharomyces* Genome Database[6] and converted to GTF format using custom Perl scripts. Different versions of RNA-analysis tools were used for the LF treatments and matched controls. These include Bowtie (version 2.2.4), TopHat (version 2.0.13) with options "-N 2 --library-type fr-unstranded" and Htseq (version 0.6.1p1) with the same options as described above.

Analysis of Differential Gene Expression

To identify the genes that were differentially expressed in *S. cerevisiae* treated with AMB, LF or AMB-LF, biological triplicate RNA-seq samples were analyzed for each treatment, with 18 samples in total. The data generated from HiSeq2000 and NextSeq platforms were analyzed separately. For the samples analyzed with HiSeq2000, there were on average 10 million reads per sample (approx. 170x coverage) and for the samples analyzed with NextSeq, there were on average 27 million reads per sample (approx. 170x coverage). Genes with low raw read counts were removed and only genes with 7 counts per million for at least 6 samples were kept for analysis. Differential expression of genes was analyzed using in R (version 3.2.5) and the edgeR library (version 3.12.0)[7]. Multiple dimension scaling was used to examine the similarity in read counts between the samples and to assess reproducibility of the replicates. The samples were normalized with respect to the library size and tag-wise biological coefficient of variation. A linear model was used to adjust for lanes effect from sequencing the samples on different sequencing lanes. Statistically significant differentially expressed genes were identified using edgeR[7]. An adjusted p-value of 0.05 was used as the cutoff for identifying differentially expressed genes. The log fold-change in gene expression that was calculated using edgeR was compared with those calculated using DESeq2[8], with high reproducibility between the two methods (Supplementary Figure S2). The results generated

using edgeR were used for all subsequent analyses as these identified a lower number of differentially expressed genes and were therefore more stringent.

For the analysis of the LF treatment, RNA-seq data were analyzed with R (version 3.2.5) and edgeR (version 3.12.0)[7] as above. Genes with low raw read counts were removed and only genes with 7 counts per million for at least 3 samples were kept for analysis. The samples were normalized with respect to the library size using the Trimmed Mean of M-values (TMM) method and the biological coefficient of variation was estimated using the tag-wise method[7]. The reproducibility of the replicates was analyzed using multidimensional scaling (Supplementary Figure S4a). Any batch effects from the experiments carried out on the different lanes were eliminated using a generalized linear model. An adjusted p-value of 0.05 was used as the cutoff for identifying differentially expressed genes. The log fold-change in gene expression calculated using edgeR. The edgeR results were compared with those obtained through DESeq2 (data not shown). No significantly differentially expressed genes were identified in the LF treatment and therefore this treatment was not subject to further analysis.

Self-Organizing Maps

Self-organizing map (SOM) analysis was used to identify clusters of differentially expressed genes with similar gene expression profiles. Transcript data from the AMB and AMB-LF treatments were first organized in two separate self-organizing maps (SOMs) to identify clusters of genes with correlated expression patterns. Several normalization steps were performed on the read counts for each sample, these included: 1) normalization with respect to the library size using the 'cpm' function in EdgeR, 2) normalization with respect to gene length to obtain the reads per kilobase per million (rpkm) value, 3) the log_2 rpkm values across the 12 samples were scaled to a mean of 0 and standard deviation of 1 per gene using Pareto scaling implemented in the 'genefilter' library (version 1.50.0).

The SOM was generated using the R 'kohonen' library (version 2.0.19)[9]. A 5 x 5 grid, the Von Neumann neighborhood function and 100 iterations of the learning algorithm were applied to each SOM. Each cluster contained genes with highly similar gene expression profiles, with neighbouring clusters having less similarity. The 5 x 5 grid was selected over other sizes (4 x 4 or 6 x 6) to identify clusters that were not too sparse or dense (data not shown).

Each self-organizing map figure was drawn using custom R scripts and the 'lattice' library (version 0.20-33). The gene expression profile for each gene consisted of the scaled values (y-axis) for the 12 samples (x-axis). The clusters were numbered from bottom to top and from left to right in the self-organizing map, such that the bottom-left cluster is number 1 and the top-right cluster is number 25. To provide a summary view of the SOM map, a heat map was used to present the average expression pattern for each cluster. The Euclidian distance function was used to assess the similarity in the gene expression profile and the 'average' agglomeration method was used to group the clusters.

Functional Enrichment Analysis

Lists of significantly differentially expressed genes from the AMB and AMB-LF treatments were separately analyzed for enrichment of Gene Ontology (GO) biological process terms using the ClueGO[10] tool. To identify active or inactive pathways, genes with increased or decreased gene expression were analyzed separately. The Bonferroni step down method was used to adjust the p-values, and GO terms with adjusted p-value < 0.05 were deemed significant. GO terms that shared a high number of genes were merged together, if the Cohen's kappa score was below 0.13, and the most significant GO term was considered representative and was shown for this analysis. The bar chart that plots the negative log p-values of each enriched GO term was drawn using Tableau visual analytic software (version 9.0).

Each cluster of co-expressed genes from the SOMs was also analyzed for enriched GO terms using the R statistical analysis package and the following libraries: 'GO.db' (version 3.2.2), 'org.Sc.sgd.db' (version 3.2.3), 'GOstats' (version 2.36.0)[11] and 'multtest' (version 2.26.0)[12]. Any GO term that mapped to one gene only was excluded from the analysis. To account for the hierarchical relationships among GO terms, a conditional hypergeometric tests was used to adjust the significance of a parent GO term based on the significance of the child GO terms. A GO term was considered to be significant if the adjusted p-value was less than 0.01.

Comparison of enriched GO terms identified prior to and after gene expression profile clustering using SOM

We compared the number of enriched GO biological process terms that were identified either before or after the application of SOM to cluster genes with similar gene expression profiles (Supplementary Figure S7). We also tallied the number of enriched GO terms that were in common, before and after clustering. The analyses described above were performed separately on lists of differentially expression genes from AMB or AMB-LF treatments, and for each treatment the genes with increased or decreased expression were also analyzed separately. We also determined if relationships between GO terms in the GO hierarchical tree affected the number of unique GO terms identified before or after clustering with SOM. Relationships among the GO terms were obtained from http://www.geneontology.org/ontology/go.obo). To compare GO terms identified before and after clustering with SOM, two GO terms were counted as non-unique if they shared ancestor-descendant relationships in the GO hierarchical tree. High-level GO terms with a depth of less than 2 were excluded from analyses of the ancestor-descendant relationships.

Network-based Enrichment of Transcription Factor Targets in Co-expressed Gene Clusters

Network-based enrichment of transcription factor gene target relations from the Yeastract[13] and YeastMine[14] databases were used to investigate whether co-expressed genes in the same SOM cluster were likely to be co-regulated by a common transcription factor[13,14]. A list of 207 known *S. cerevisiae* transcription factors was obtained from the literature[15,16]. To check whether these transcription factors were responsible for the regulation of gene co-expression, each cluster was analyzed for an enrichment of targets for any of the known transcription factors. The result consisted of a list of transcription factors that have high probability of regulating the expression of the known target genes in the cluster. To increase the specificity of the transcription factors identified, two different approaches were used and the results that were in common between the two approaches were reported. The first approach involved the use of Yeastract[13] (version 2013-09-27), a database of curated transcription factor and target gene interactions. Fisher's exact test was used to calculate the probability of enrichment and the p-values were adjusted for false discovery rate. A transcription factor was considered to be enriched if the adjusted p-value was < 0.0001. For each cluster, any transcription factors with one or more target genes in the cluster were analyzed. The second approach used the YeastMine publication enrichment tool[14]. Each cluster of co-expressed genes was analyzed separately for an over-representation of known transcription factor and target genes relationships from the literature. Significant hits with Benjamini-Hochberg adjusted p-value of < 0.05 were filtered by searching for literature with a title containing the gene name of known transcription factors. For the two approaches, the background set included the total set of yeast genes in the SGD database.

Visualization of Integrated Transcription Factor-Target Gene Networks and Protein Interaction Networks

To investigate transcription factor-target interactions and the protein-protein interactions between transcription factors and co-regulatory factors, an integrated network was constructed. This network was created from the union of the Yeastract[13] transcriptional regulatory network (version 2013-09-27). and the protein interaction network from Pang *et al.* (2012)[17]. Genes involved in iron uptake were as curated by Philpott and Protchenko (2008)[18] and genes involved in zinc homeostasis were as curated by Wu *et al.* (2008)[19] and Eide (2009)[20]. The integrated networks were visualized using Cytoscape version 3.1.1[21]. Log fold-changes from differential expression analysis were co-visualized as node colors.

Apoptosis and oxidative stress response genes

Genes involved in oxidative stress were identified from the *Saccharomyces* Genome Database (SGD)[6] by matching the key word “oxidative stress” against the gene description field or the Gene Ontology Biological Process terms for that gene. Similarly, a list of genes involved in apoptosis was identified with the key word “apoptosis”. Additional genes involved in apoptosis from Carmona-Gutierrez *et al.* (2010)[22] and Belenky *et al.* (2013)[23] were also added to our list.

Selection of genes for chemical-genetic experiments

A list of criteria was used to select query genes to perform chemical-genetic experiments with gene knockout mutants. The query genes were selected from the list of genes with significant differential expression in AMB or AMB-LF treatments. We narrowed the list further to genes that were representative of enriched biological processes involved in response to stress or drug treatments or were targets of transcription factors Aft1p and Zap1, as annotated in Yeastract[13]. Additional information was included to identify putative drug targets. Using OrthoMCL[24], we checked whether the query *S. cerevisiae* genes had orthologs in four fungal pathogens (*C. neoformans*, *C. albicans*, *C. glabrata* and *A. fumigatus*) and in humans. We also used reciprocal BLAST[25] searches to compare the proteomes of *C.*

neoformans and *S. cerevisiae*, and reciprocal best hits are also identified as orthologs. We also checked whether deletion of the query gene was known to increase sensitivity to AMB (148 nM or 100 μM) in a large-scale chemical-genetic screen[26], and whether the *S. cerevisiae* proteins of interest shared common protein domains (Pfam version 27.0)[27] with 'druggable' proteins from the DrugBank database (version 4.1)[28].

***Saccharomyces cerevisiae* haploid single gene knockout mutants**

Knockout mutants were obtained from the Yeast Deletion Project[29] for the *S. cerevisiae* MATa BY4741 strain. Knockouts were confirmed using PCR as previously described by the Yeast Deletion Project (http://sequence-www.stanford.edu/group/yeast_deletion_project/confirmation.html). Briefly, the sequences around the knockout site were amplified using 2.5 U of Taq polymerase (NEB), 0.2 mM dNTPs, 1 μM of upstream primer (A, KanC), 1 μM downstream primer (B, KanD, D), 10 ng genomic DNA in 50 μL volumes. A list of the primer combinations (A-B, A-KanB, KanC-D and A-D) designed for verifying each mutant and wild type strain are included in Supplementary Table S5. Amplification conditions were 94 °C for 3 min, 35 cycles of 94 °C for 15 sec, 57 °C for 15 sec and 72 °C for 60 sec, and 72 °C for 3 min. Primer combinations A-B, A-KanB, KanC-D and A-D were designed for each selected gene and for the wild type/background strain. Amplification of bands from primers A-KanB and KanC-D were required for knockout confirmation. Where bands were not present with either primer combinations A-KanB or KanC-D, amplifications from primers A-D sufficed. Additionally, primers A-B were used to amplify DNA sequences from the wild type strain BY4741 to confirm gene identity[30].

***Saccharomyces cerevisiae* gene knockout design (YOR387C)**

Gene knockouts were performed using a modified protocol from Janke *et. al*. (2004)[31]. Plasmid cassettes pFA6a-natNT2 and pFA6a-hphNT1 were used to generate a *yor387cΔ* knockout mutant with BY4741 and a *yor387cΔ/vel1Δ* double knockout from the *vel1Δ* knockout mutant. Primer sequences 45-55 bp before and after and inclusive of the start and stop codon of YOR387C were designed and tagged with a common sequence homologous to the marker cassette within the plasmid at the 3' end. The primers designed were: S1 – 5' ttctgatagattgtacaatctcaagaaatcaagaacaacaaccataccatgcgtacgctgcaggtcgac 3'. S2 – 5' taaaaaatatacttaaaatatgtctacagattatgcagctggaaaaaatcaatcgatgaattcgagctcg 3'. Gene deletion constructs were amplified with 2 x Long amp Taq master mix (NEB), 10 µM each of S1 and S2 primers, 10 ng plasmid cassette DNA in 50 µL volumes. Constructs were denatured at 94 °C for 3 min followed by 30 cycles of 94 °C for 30 sec, 54 °C for 30 sec and 65 °C for 4 min. The final extension was at 65 °C for 10 min.

Transformation

Five µL of the constructed plasmid cassette DNA was transformed into the wild type BY4741 and *vel1* knockout mutant (KanMX4 marker, BY4741 background) using the lithium acetate protocol with slight changes (http://research.fhcrc.org/gottschling/en/protocols/yeast-protocols/transformation.html)[32]. Briefly, an overnight YPD cultures of BY4741 was sub-cultured into 50 mL of pre-warmed YPD broth at a starting concentration of 5 x 10^6 cells/mL. The culture was grown at 30 °C, 200 rpm for at least two rounds of cell division before harvesting at 4000 rpm for 5 min. Cells were then washed and resuspended in 100 mM lithium acetate (LiAc) to a final cell concentration of 2 x 10^9 cells/mL in 500 µL. 50 µL of the cell suspension with LiAc aspirated was used for transformation. The transformation reagents were added in the following order to a total volume of 360 µL: 240 µL PEG (50% w/v), 36 µL 1 M LiAc, 25 µL of pre-boiled and chilled SS-DNA (4 mg/mL), no more than 36 µL plasmid DNA and sterile

distilled water. The cells and reagents were vortexed until mixed before incubation at 30 °C for 30 min and then heat-shocked for an additional 30 min at 42 °C. Transformed cells were pelleted by centrifugation at 7,000 rpm for 90 sec and incubated overnight at room temperature in SDB before mutant selection on SDA supplemented with nourseothricin (100 mg/L) at 30 °C for up to 5 days. Knockout mutants were confirmed with the following primers: KO_yor387cA – 5’ ttcggatctttcctaaacgg 3’, KO_yor387cC – 5’ actcttgggcagacaccg 3’, KO_yor387cD – 5’ tgacaagaaaaccatctgcg 3’, NatB – 5’ attcgtcgtccgattcgtc 3’, NatC – 5’ tacatgagcatgccctgc 3’.

Analysis of knockout mutants using spot dilution assays

Spot dilution assays were performed on synthetic complete (SC) media (1.71 g/L yeast nitrogen base (BD), 5g/L ammonium sulphate, 20 g/L glucose, 1.92 g/L SD-ura drop out (sigma), 76 mg/L uracil (sigma), 20 g/L agar). Overnight SC broth cultures of knockout mutants and BY4741 were grown at 30 °C, 180 rpm. Unless specified otherwise, cell concentrations were standardized at 1 x 10^6 cells/mL with a haemocytometer and 1:10 dilutions were made, ranging from 10^6 to 10 cells/mL. Five µL aliquots were spotted onto plates supplemented with stressing agents at the following concentration ranges: AMB; 0.25 – 1 µg/mL, fluconazole (FLC); 16 – 64 µg/mL, calcofluor white (CW); 100 – 200 µg/mL, SDS; 0.005 – 0.02 µg/mL, NaCl; 1 M, $NaNO_2$; 1 – 3 mM, H_2O_2; 1 – 3 mM, caffeine; 10 – 15 mM, 37 °C. Plates were left to dry and incubated at 30 °C, checking cell growth every day until the 5th day.

Supplementary Results

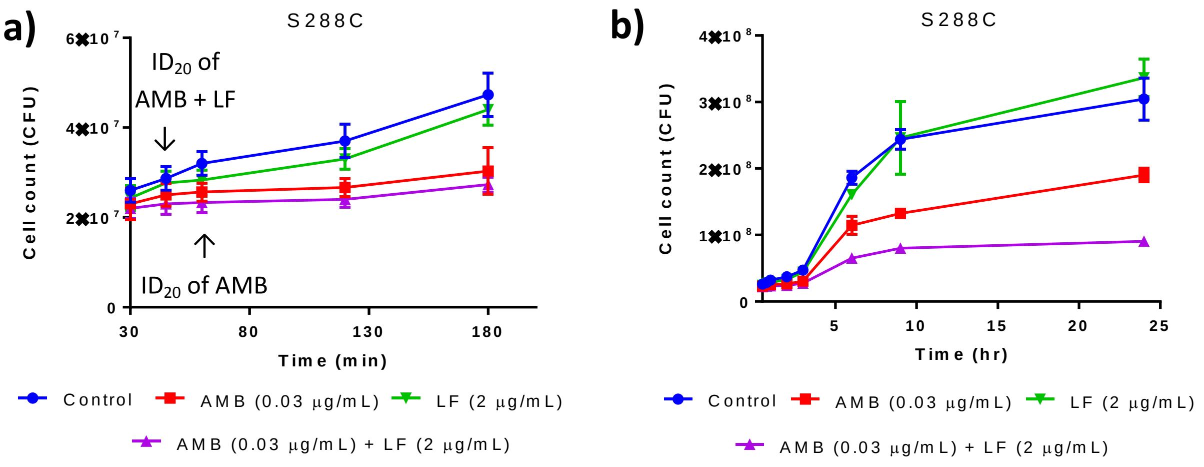

Supplementary Figure S1: The effect of AMB-LF, AMB and LF on the growth of *S. cerevisiae*. a) First 3 hours of treatment. The ID_{20} is 50 minutes for AMB-LF treatment and 1 hour for AMB treatment. **b)** Growth curves extended for 24 hours. The growth in AMB and AMB-LF treatments indicate a fungistatic response. The LF at 2ug/mL did not inhibit S288C growth throughout 24 hours of treatment. Data shown as mean +/- standard error of the mean (SEM)**.**

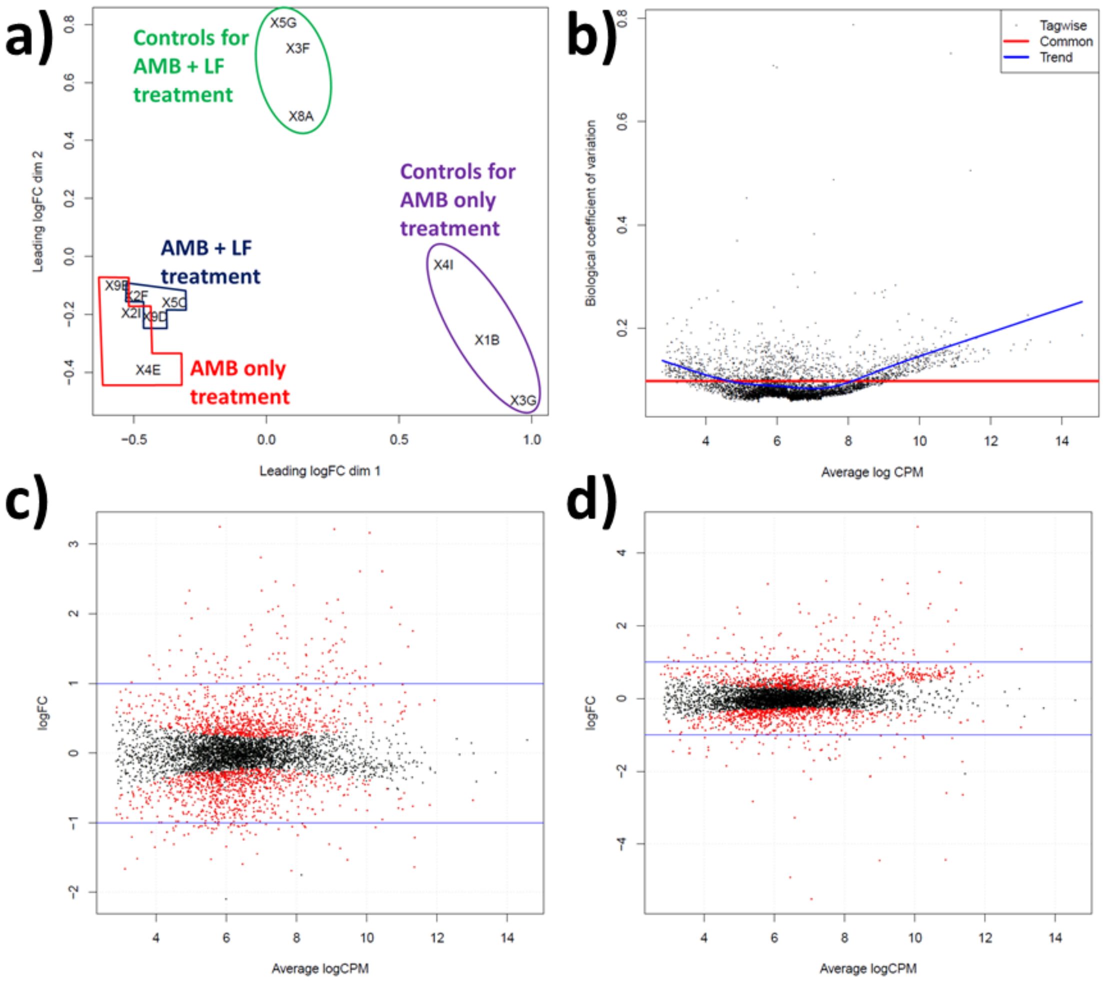

Supplementary Figure S2: RNA-seq analyses of the AMB and AMB-LF treatments. a) Multiple dimensional scaling (MDS) plot of the RNA-seq samples for the AMB and AMB-LF treatments and their matched controls. The gene expression profiles for the 12 samples were normalized with respect to library size using edgeR and visualized. Biological replicates have similar gene expression profiles and are grouped closer together . AMB and AMB-LF treatments have similar gene expression profiles, but the two control samples have greater differences. **b)** The plot of the biological coefficient of variation (y-axis) versus average $\log_2$ counts per million (CPM; x-axis) for the RNA-samples shown in **a)**. The average biological coefficient of variation is 0.098. The red line represents the common dispersion for all genes, the blue line represents the trended dispersion, and each data point represents the tag-wise dispersion for each gene. The smear plots display the $\log_2$-fold change versus the average $\log_2$ CPM value of each gene for **c)** the AMB treatment and **d)** the AMB-LF treatment. Differentially expressed genes are highlighted in red and the blue lines are drawn at two-fold increase or decrease in gene expression.

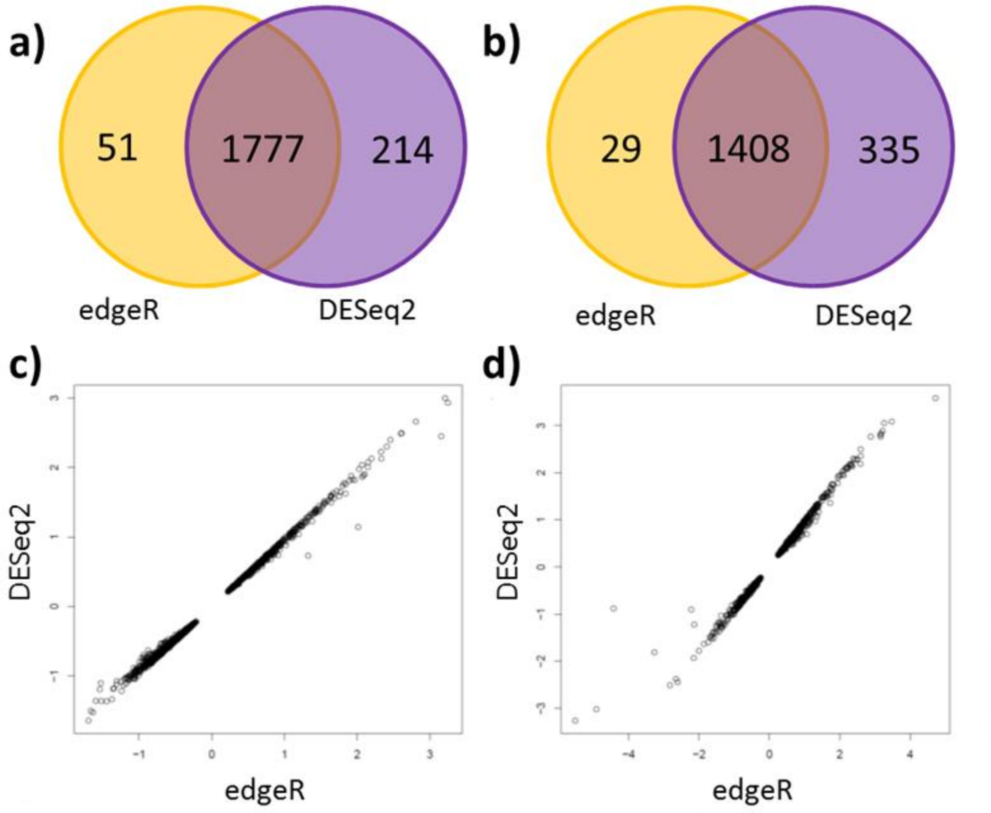

Supplementary Figure S3: Comparison of differentially expressed genes called by the edgeR and DESeq2 RNA-Seq analysis tools. Venn diagram comparing the number of differentially expressed genes called by edgeR and DESeq2 for the **a)** AMB and **b)** AMB-LF treatments. Scatter plots were used to compare the $\log_2$ fold-change of the differentially expressed genes commonly identified by DESeq2 (y-axis) and edgeR (x-axis) for the **c)** AMB and **d)** AMB-LF treatments. Pearson correlation values of 0.998 and 0.985 were observed **c)** and d**)** respectively (p-values < 0.01). Both the Venn diagram and scatter plots suggest a high degree of concordance between edgeR and DESeq2, suggesting the RNA-seq data analysis is consistent between tools. EdgeR was more stringent for the analyses performed here as it consistently resulted in lower number of significant differentially expressed genes.

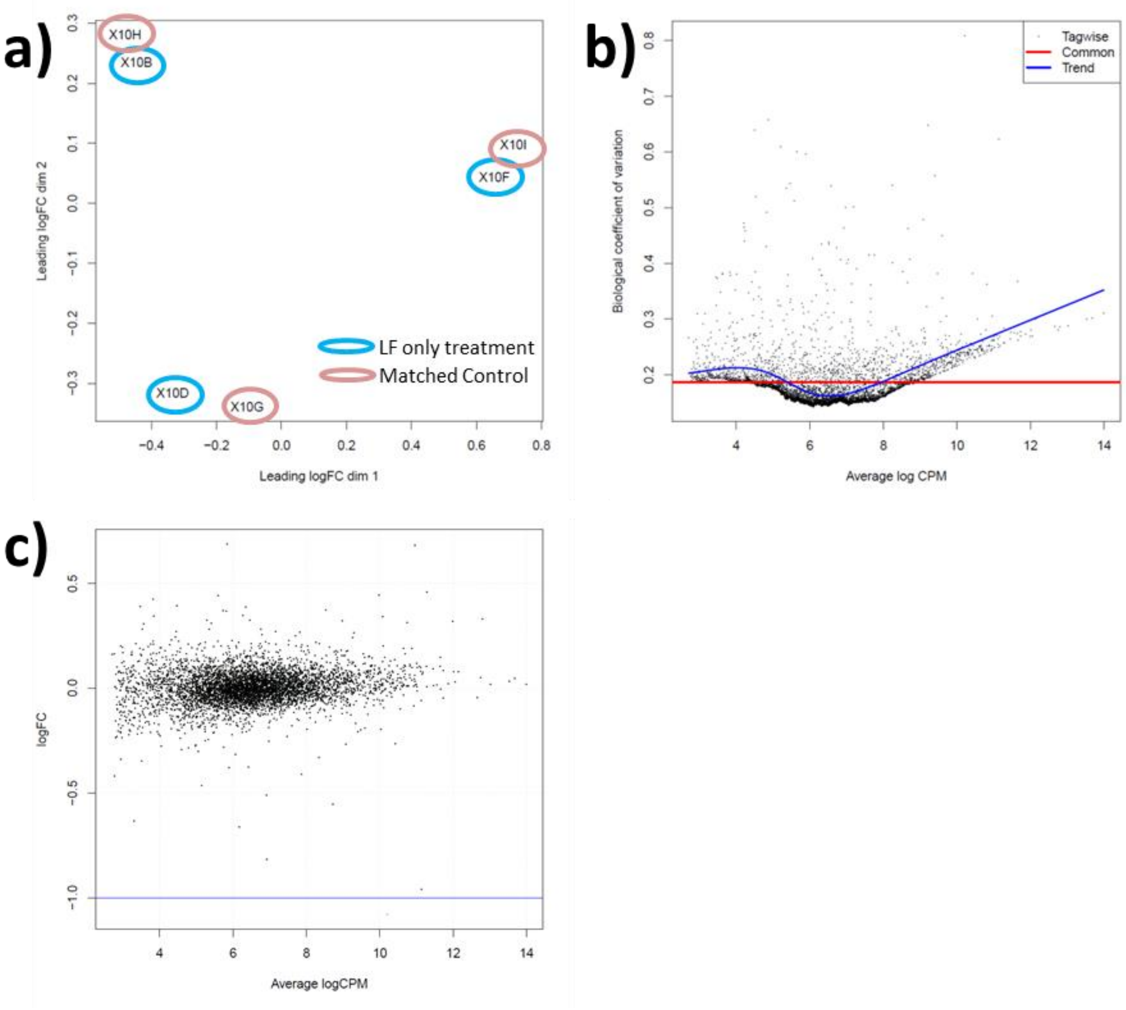

Supplementary Figure S4: RNA-seq analyses of the LF-only treatment. a) Multiple dimensional scaling (MDS) plot of the RNA-seq samples for the LF treatment and the corresponding matched controls. The gene expression profiles for the 6 samples were normalized with respect to library size using edgeR and visualized using MDS. The three clusters of samples represent three biological replicates performed on three different days. **b)** The biological coefficient of variation (y-axis) versus average $\log_2$ CPM (x-axis) for the RNA-samples shown in **a)**. The average biological coefficient of variation is 0.187. The red line represents the common dispersion for all the genes, the blue line represents the trended dispersion, and each data point represents the tag-wise dispersion for each gene. **c)** The smear plot displays the $\log_2$ fold-change versus the average $\log_2$ CPM value of each gene for the LF-only treatment. The blue line is drawn at two-fold change decrease in gene expression but there were no differentially expressed up- or down-regulated genes.

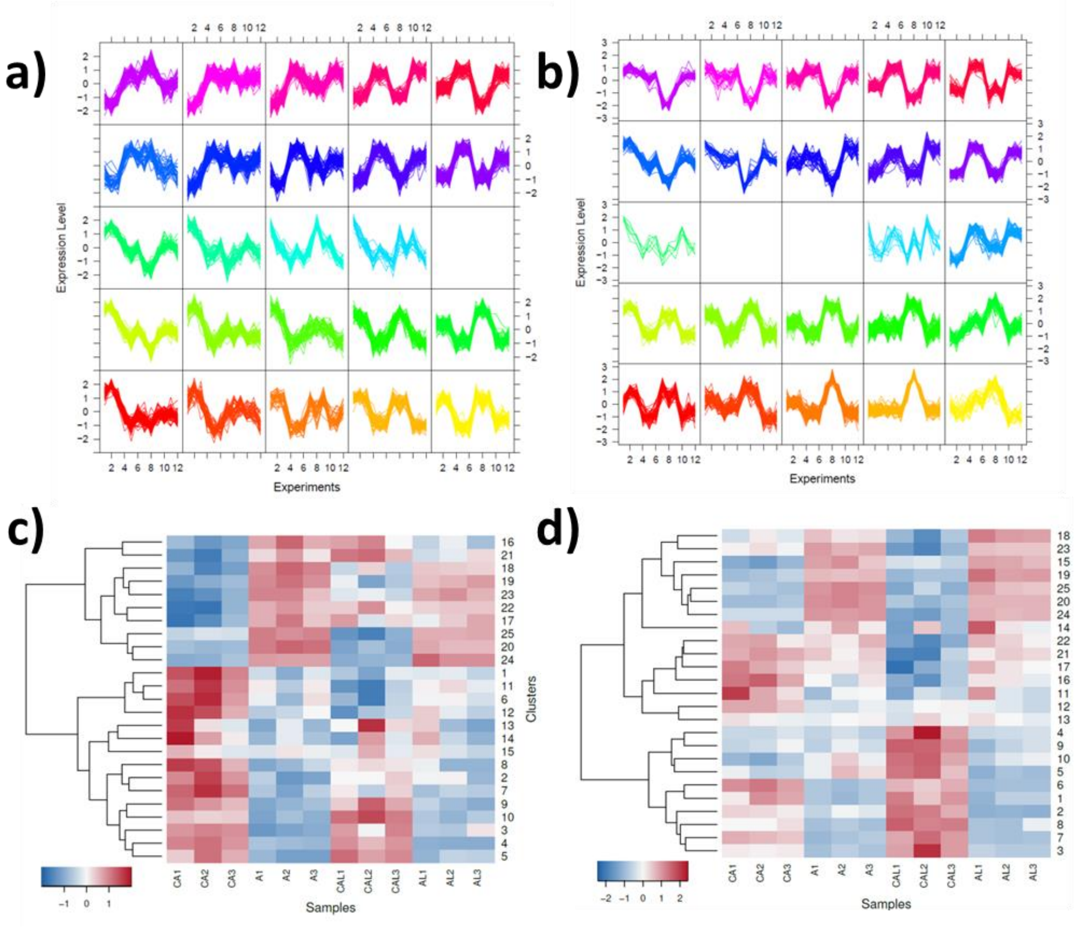

Supplementary Figure S5: Self-organizing maps (SOMs) for the AMB and AMB-LF treatments. Complete SOMs for the **a)** AMB and **b)** AMB-LF treatments. Gene expression profiles (in $\log_2$ rpkm) across the 12 samples for each gene were scaled to a mean of 0 and a standard deviation of 1, with respect to the mean and standard deviation values for each gene (Pareto scaling). In the SOM cluster, each differentially expressed gene is represented by a line and the y-axis represent the scaled gene expression across the 12 experimental samples along the x-axis. Each cluster in the 5 x 5 map represents a group of genes with similar gene expression profile. The 12 samples were arranged from left to right, starting with three matched controls for AMB treatment, three samples for AMB treatment, three control samples for the AMB-LF treatment, and three samples for the AMB-LF treatment. Heat maps of average gene expression for each SOM cluster for **c)** AMB and **d)** AMB-LF treatments. The color scale is shown at the bottom left of the panels; red represents increased gene expression and blue represents decreased gene expression. The y-axis represents the 25 clusters and the x-axis represents the 12 samples. The four treatment types are labelled in the x-axis with the biological triplicates numbered; A = AMB, AL = AMB-LF, CA = matched control for AMB treatment, CAL = matched control for AMB-LF. The dendrogram on the left divide the clusters into two large groups, which contains up- or down-regulated genes exclusively. **c)** For the AMB treatment, up-regulated genes were found in clusters 16-25, down-regulated genes were found in clusters 1-15. **d)** For the AMB-LF treatment, up-regulated genes were found in clusters 11-25, down-regulated genes were found in clusters 1-10.

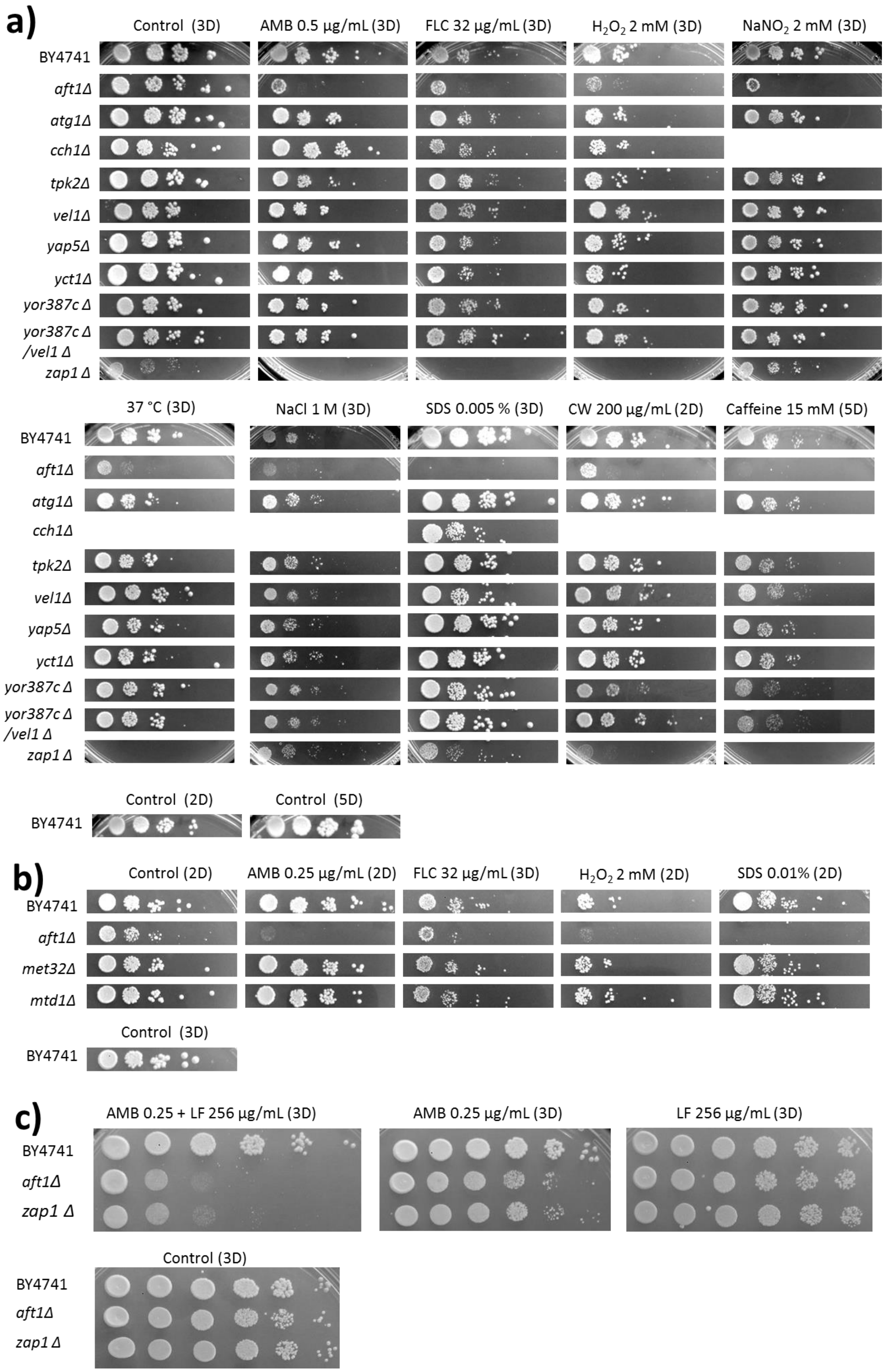
a)
Control (3D)
AMB 0.5 µg/mL (3D)
FLC 32 µg/mL (3D)
H_2O_2 2 mM (3D)
$NaNO_2$ 2 mM (3D)
BY4741
aft1Δ
atg1Δ
cch1Δ
tpk2Δ
vel1Δ
yap5Δ
yct1Δ
yor387c Δ
yor387c Δ /vel1 Δ
zap1 Δ
37 °C (3D)
NaCl 1 M (3D)
SDS 0.005 % (3D)
CW 200 µg/mL (2D)
Caffeine 15 mM (5D)
BY4741
aft1Δ
atg1Δ
cch1Δ
tpk2Δ
vel1Δ
yap5Δ
yct1Δ
yor387c Δ
yor387c Δ /vel1 Δ
zap1 Δ
Control (2D)
Control (5D)
BY4741
b)
Control (2D)
AMB 0.25 µg/mL (2D)
FLC 32 µg/mL (3D)
H_2O_2 2 mM (2D)
SDS 0.01% (2D)
BY4741
aft1Δ
met32Δ
mtd1Δ
Control (3D)
BY4741
c)
AMB 0.25 + LF 256 µg/mL (3D)
AMB 0.25 µg/mL (3D)
LF 256 µg/mL (3D)
BY4741
aft1Δ
zap1 Δ
Control (3D)
BY4741
aft1Δ
zap1 Δ

Supplementary Figure S6: The *aft1Δ* and *zap1Δ* mutants showed increased susceptibility to multiple stressors. The gene deletion mutants **a)** *aft1Δ*, *atg1Δ*, *cch1Δ*, *tpk2Δ*, vel1*Δ*, *yap5Δ*, *yct1Δ*, yor387c*Δ,* vel1*Δ*/yor387c*Δ* and *zap1Δ*, **b)** *aft1Δ*, *met32Δ*, *mtd1Δ,* were compared to the *S. cerevisiae* wild type (BY4741) background. Ten-fold serial dilutions of the strains were plated on synthetic complete agar plates from left to right starting at 10^6 cells/mL with different types of stressors. 'D' indicates the days of growth at 30 °C. Stressors tested include antifungal drugs (amphotericin B (AMB), fluconazole (FLC)), oxidative stress (H_2O_2), nitrosative stress ($NaNO_2$), heat stress (37 °C) and cell wall stressing agents (NaCl, SDS, calcofluor white (CW) and caffeine). CW was tested at 200 µg/mL except for *vel1*, *yor387c* and *vel1/yor387c* where 300 µg/mL CW was used. In (a) c*ch1* was tested later with only selected stressing agents. Only *aft1Δ* and *zap1Δ* mutants showed increased sensitivity to AMB and other types of stressors. These suggest Aft1p and Zap1p are important for resistance to AMB and oxidative stress. The *aft1Δ* strain had increased susceptibility to calcofluor white, NaCl, $NaNO_2$, SDS and caffeine, and the *zap1Δ* strain was susceptible to all stressors except SDS, $NaNO_2$ and NaCl. These results indicate Aft1p and Zap1p are important in stress responses in general. **c)** The *aft1Δ* and *zap1Δ* mutants were further tested with AMB in combination with LF. Both *aft1Δ* and *zap1Δ* showed growth retardation in the presence of AMB-LF but not to the individual agents alone.

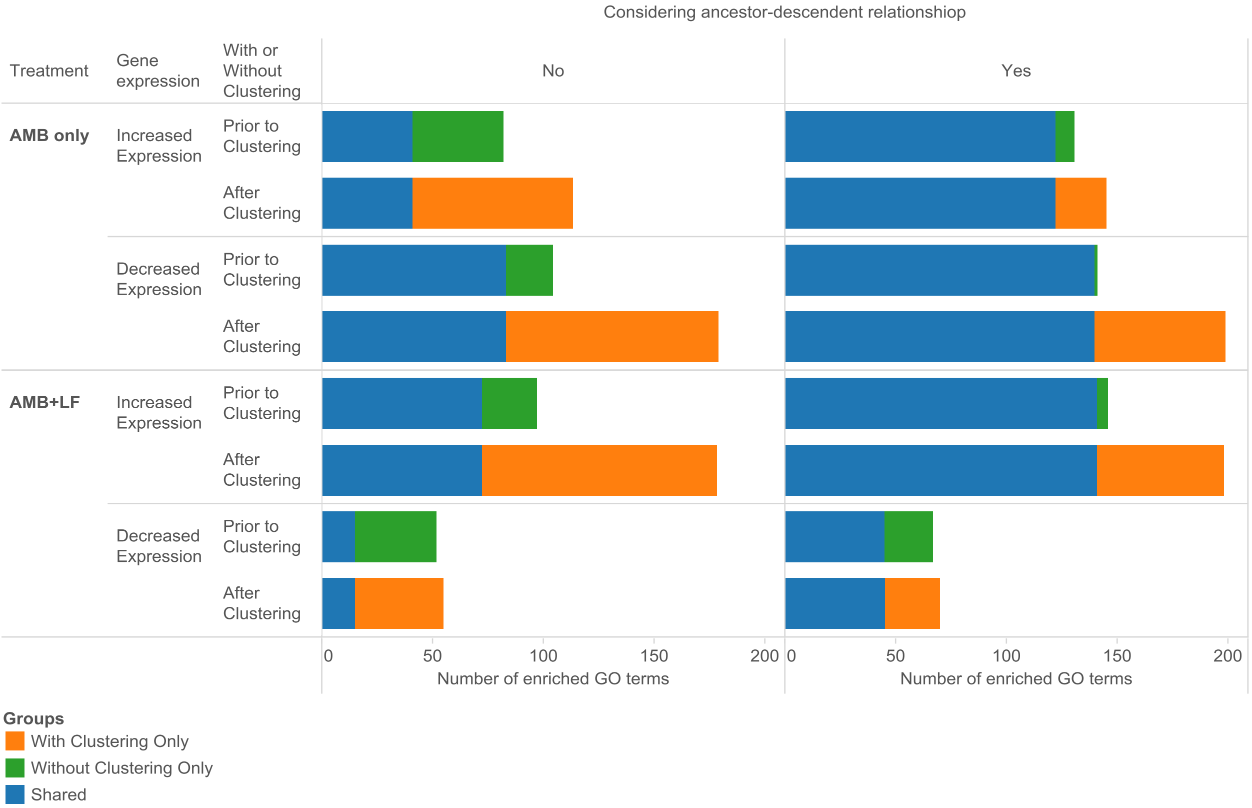

Supplementary Figure S7: The numbers of enriched GO biological processes terms identified prior to and after SOM clustering. Results were divided into groups based on the type of treatment, increased or decreased gene expression and whether clustering had been applied prior to GO term enrichment

(graph on the left hand side). The horizontal bars show the number of enriched GO terms identified prior to clustering analysis (green bar), after clustering analysis (orange bar) or shared by both analyses (blue bar). To demonstrate that the increased percentage is due to identification of GO terms unique to the clustering analysis, we reanalyzed the data and GO terms were considered to be unique only if related ancestor-descendent relationships were not identified prior to clustering (graph on right hand side). We consistently observed a higher number of enriched GO terms after clustering analysis.

Supplementary Table S1: Design of experiments to analyze the transcriptomic profiles of *S. cerevisiae* treated with AMB, LF or AMB-LF.

Experiment	AMB concentration	LF concentration	ID_{20} time point (mins.)
AMB	0.03 µg/mL	0	60
Control for AMB	0	0	60
AMB-LF	0.03 µg/mL	2 µg/mL	50
Control for AMB-LF	0	0	50
LF	0	2 µg/mL	50
Control for LF	0	0	50

Supplementary Table S2: List of Genes Involved in Apoptosis

Standard Name	Systematic Name	Name Description[a]	AMB treatment[b]	AMB-LF treatment[b]
TPK1	YJL164C	Takashi's Protein Kinase	0.52	0.00
RNY1	YPL123C	RiboNuclease from Yeast	0.49	0.00
PEP4	YPL154C	carboxyPEPtidase Y-deficient	0.44	0.00
TPK2	YPL203W	Takashi's Protein Kinase	0.43	0.00
SDH1	YKL148C	Succinate DeHydrogenase	0.37	0.00
FYV10	YIL097W	Function required for Yeast Viability (plays role in anti-apoptosis)	0.34	0.00
WWM1	YFL010C	WW domain containing protein interacting with Metacaspase	0.32	0.00
BMH2	YDR099W	Brain Modulosignalin Homologue	0.32	0.00
NDI1	YML120C	NADH Dehydrogenase Internal	0.32	0.00
CYR1	YJL005W	CYclic AMP Requirement	0.31	0.00
CIT1	YNR001C	CITrate synthase	0.30	0.00
RAS2	YNL098C	homologous to RAS proto-oncogene	0.29	0.00
SDH2	YLL041C	Succinate DeHydrogenase	0.29	0.00
GDH2	YDL215C	Glutamate DeHydrogenase	0.29	0.00

Standard Name	Systematic Name	Name Description[a]	AMB treatment[b]	AMB-LF treatment[b]
		(suppresses apoptosis)		
AIF1	YNR074C	Apoptosis-Inducing Factor	0.29	0.00
DRE2	YKR071C	Derepressed for Ribosomal protein S14 Expression	0.00	0.29
GCG1	YER163C	Gamma-glutamyl Cyclotransferase acting on Glutathione	0.00	-0.35
LOT6	YLR011W	LOw Temperature-responsive	0.00	-0.66
PDE2	YOR360C	PhosphoDiEsterase	0.00	0.50
STM1	YLR150W	Suppressor of ToM1	0.00	0.53
STE20	YHL007C	STErile	-0.31	0.00
OYE2	YHR179W	Old Yellow Enzyme	-0.36	-0.47
IZH2	YOL002C	Implicated in Zinc Homeostasis	-0.36	0.00
TIF3	YPR163C	Translation Initiation Factor	-0.37	0.00
SXM1	YDR395W	Suppressor of mRNA eXport Mutant	-0.39	0.00
NUC1	YJL208C	NUClease	-0.45	0.00
NMA111	YNL123W	Nuclear Mediator of Apoptosis	-0.55	0.00
RAS1	YOR101W	homologous to RAS proto-oncogene	-0.58	0.00
TDH2	YJR009C	Triose-phosphate DeHydrogenase	-1.63	-1.74

a) Name description from the Saccharomyces Genome Database.

b) Red indicates up-regulation and green indicates down-regulation of gene expression. More extreme levels of fold-changes have darker shades. Values represent fold-change in log base 2.

Supplementary Table S3: List of Genes Involved in Oxidative Stress

Standard Name	Systematic Name	Name Description[a]	AMB treatment[b]	AMB-LF treatment[b]
**A. Oxidative stress genes with an opposing direction of fold-change (n=7)**				
HSP12	YFL014W	Heat Shock Protein	0.69	-0.59
MCR1	YKL150W	Mitochondrial NADH-Cytochrome b5 Reductase	0.32	-0.41
HYR1	YIR037W	HYdroperoxide Resistance	0.26	-0.32
RLI1	YDR091C	RNase L Inhibitor	-0.41	0.31
YHB1	YGR234W	Yeast flavoHemogloBin	-0.54	0.73
GPX2	YBR244W	Glutathione PeroXidase	-0.76	0.42
LTV1	YKL143W	Low Temperature Viability	-0.97	0.67

Standard Name	Systematic Name	Name Description[a]	AMB treatment[b]	AMB-LF treatment[b]
**B. Oxidative stress genes differentially expressed in one treatment only (n=42)**				
CTT1	YGR088W	CaTalase T	1.02	0.00
NCE103	YNL036W	NonClassical Export	0.77	0.00
XBP1	YIL101C	XhoI site-Binding Protein	0.74	0.00
ZTA1	YBR046C	ZeTA-crystallin	0.70	0.00
UGA2	YBR006W	Utilization of GAba	0.70	0.00
HSP31	YDR533C	Heat-Shock Protein	0.68	0.00
TPS1	YBR126C	Trehalose-6-Phosphate Synthase	0.67	0.00
GAD1	YMR250W	GlutAmate Decarboxylase	0.60	0.00
ZWF1	YNL241C	ZWischenFerment	0.60	0.00
ASK10	YGR097W	Activator of SKn7	0.49	0.00
HSP150	YJL159W	Heat Shock Protein	0.45	0.00
PST2	YDR032C	Protoplasts-SecreTed	0.38	0.00
DOT5	YIL010W	Disruptor Of Telomeric silencing	0.33	0.00
GRX2	YDR513W	GlutaRedoXin	0.31	0.00
UBP2	YOR124C	UBiquitin-specific Protease	0.29	0.00
GSH2	YOL049W	Glutathione	0.27	0.00
RPL22A	YLR061W	Ribosomal Protein of the Large subunit	0.00	0.64
TAH18	YPR048W	Top1T722A mutant Hypersensitive	0.00	0.63
SCH9	YHR205W		0.00	0.32
MHR1	YDR296W	Mitochondrial Homologous Recombination	0.00	-0.30
GET3	YDL100C	Guided Entry of Tail-anchored proteins	0.00	-0.34
GCY1	YOR120W	Galactose-inducible Crystallin-like Yeast protein	0.00	-0.40
GRX7	YBR014C	GlutaRedoXin	0.00	-0.41
GEX2	YKR106W	Glutathione EXchanger	0.00	-0.45
GRX1	YCL035C	GlutaRedoXin	0.00	-0.52
GPX1	YKL026C	Glutathione PeroXidase	0.00	-0.55
GRE3	YHR104W	Genes de Respuesta a Estres (stress responsive genes)	0.00	-0.65
LOT6	YLR011W	LOw Temperature-responsive	0.00	-0.66
TSA1	YML028W	Thiol-Specific Antioxidant	0.00	-0.66
HMX1	YLR205C	HeMe oXygenase	0.00	-0.83
TSA2	YDR453C	Thiol-Specific Antioxidant	0.00	-0.86
PRX1	YBL064C	PeroxiRedoXin	0.00	-1.00
MDL1	YLR188W	MultiDrug resistance-Like	-0.22	0.00

Standard Name	Systematic Name	Name Description[a]	AMB treatment[b]	AMB-LF treatment[b]
AIM45	YPR004C	Altered Inheritance rate of Mitochondria	-0.25	0.00
GND1	YHR183W		-0.32	0.00
NCL1	YBL024W	NuCLear protein	-0.34	0.00
ROX1	YPR065W	Regulation by OXygen	-0.34	0.00
FAP7	YDL166C	Factor Activating Pos9	-0.39	0.00
YAR1	YPL239W	Yeast Ankyrin Repeat	-0.39	0.00
URM1	YIL008W	Ubiquitin Related Modifier	-0.62	0.00
ATX1	YNL259C	AnTioXidant	-0.68	0.00
TMA19	YKL056C	Translation Machinery Associated	-0.95	0.00
**C. Genes with similar fold-change in both treatments (n=23)**				
RCK1	YGL158W	Radiation sensitivity Complementing Kinase	1.94	2.34
GRX4	YER174C	GlutaRedoXin	1.53	1.35
TPO1	YLL028W	Transporter of POlyamines	1.13	0.94
CIN5	YOR028C	Chromosome INstability	1.11	0.95
POS5	YPL188W	PerOxide Sensitive	0.90	0.60
MTL1	YGR023W	Mid-Two Like	0.73	0.57
GTO1	YGR154C	Glutathione Transferase Omega-like	0.58	0.35
NAR1	YNL240C	Nuclear Architecture Related	0.56	0.55
ERV1	YGR029W	Essential for Respiration and Viability	0.56	0.42
SOD1	YJR104C	SuperOxide Dismutase	0.49	0.50
CCP1	YKR066C	Cytochrome c Peroxidase	0.48	0.45
MSN4	YKL062W	Multicopy suppressor of SNF1 mutation	0.41	0.65
CTA1	YDR256C	CaTalase A	0.34	0.48
EOS1	YNL080C	ER-localized and Oxidants Sensitive	0.23	0.39
CSR1	YLR380W	Chs5 Spa2 Rescue	-0.27	-0.27
GRX3	YDR098C	GlutaRedoXin	-0.31	-0.46
RPN11	YFR004W	Regulatory Particle Non-ATPase	-0.32	-0.45
OYE2	YHR179W	Old Yellow Enzyme	-0.36	-0.47
MXR2	YCL033C	peptide Methionine sulfoXide Reductase	-0.41	-0.47
SNT2	YGL131C		-0.47	-0.57
AHP1	YLR109W	Alkyl HydroPeroxide reductase	-0.58	-1.09
GRX8	YLR364W	GlutaRedoXin	-0.72	-0.70
MXR1	YER042W	peptide Methionine sulfoXide Reductase	-0.79	-1.20

a) Name description from the Saccharomyces Genome Database.
b) Red indicates up-regulation and green cindicates down-regulation of gene expression. More extreme levels of fold-changes have darker shades. Values represent fold-change in log base 2.

Supplementary Table S4: Transcription factors with an over-representation of target genes among clusters of co-expressed genes.

Gene Name	OLN	Description[a]	Yeastract adjusted p-value[b]	Yeastmine adjusted p-value[c]	Cluster ID
**AMB treatment**					
AFT2	YPL202C	Iron-regulated transcriptional activator	< 0.01	< 0.01	AMB, 17
DAL80	YKR034W	Negative regulator of genes in multiple nitrogen degradation pathways	< 0.01	< 0.01	AMB, 24
GAT1	YFL021W	Transcriptional activator of nitrogen catabolite repression genes	< 0.01	< 0.01	AMB, 24
GLN3	YER040W	Transcriptional activator of genes regulated by nitrogen catabolite repression	< 0.01	0.01	AMB, 24
**AMB-LF treatment**					
ABF1	YKL112W	DNA binding protein with possible chromatin-reorganizing activity	< 0.01	0.03	AMB-LF, 24
BAS1	YKR099W	Transcription factor involved in regulating purine and histidine biosynthesis pathways	< 0.01	0.03	AMB-LF, 24
GCN4	YEL009C	bZIP transcriptional activator of amino acid biosynthetic genes	< 0.01	< 0.01	AMB-LF, 24
RAP1	YNL216W	Essential DNA-binding transcription regulator that binds many loci	< 0.01	< 0.01	AMB-LF, 17
RPN4	YDL020C	Transcription factor that stimulates expression of proteasome genes	< 0.01	< 0.01	AMB-LF, 24
SOK2	YMR016C	Nuclear protein that negatively regulates pseudohyphal differentiation	< 0.01	0.04	AMB-LF, 24
STP1	YDR463W	Transcription factor that activates transcription of amino acid permease genes	< 0.01	0.03	AMB-LF, 24
ZAP1	YJL056C	Zinc-regulated transcription factor	< 0.01	0.03	AMB-LF,

Gene Name	OLN	Description[a]	Yeastract adjusted p-value[b]	Yeastmine adjusted p-value[c]	Cluster ID
					10

a) Descriptions from the *Saccharomyces* Genome Database
b) Transcriptional regulatory network from the Yeastract database (adj. p-value < 0.01)
c) Transcription factor and target genes relationships from the Yeastmine database (adj. p-value < 0.05)

Supplementary Table S5: List of primers used to construct and confirm knockout mutants

Primer type	5' – 3' sequence
KanB	CTGCAGCGAGGAGCCGTAAT
KanC	TGATTTTGATGACGAGCGTAAT
NatB*	ATTCGTCGTCCGATTCGTC
NatC*	TACATGAGCATGCCCTGC
*aft1*_A	AGCAGAAACAGAATTCGCATATTAC
*aft1*_B	CTTATCTTCAAAGTTGGGTACTGGA
*aft1*_D	CAAAATTAATGACAGAGGGAGAGAA
*atg1*_A	AAGTTAAGTACCAAGGCCATCTTTT
*atg1*_B	TAAAATGGGTAAGTTGTAGATCCCA
*atg1*_D	TATAGCCAAAGGCAAGTACTAAACG
*cch1*_A	AGAAAATGTAATTTGGCATGTCATT
*cch1*_B	GCTATAACTACTGAAGCTACGCCTG
*cch1*_D	AATTCCCTTCTAATGGGTACTCTTG
*met32*_A	CGCTTAGTACGCCACAGTTTATATT
*met32*_B	GCGTTTTCCTTTTTAACCTTTATGT
*met32*_D	CCAAGAACTTGAGTATTTGACAGGT
*mtd1*_A	TATTCTTTTCCTAGTCCAAGTCCTG

*mtd1*_B	CTATGCATTTCTTCCCATACAGTCT
*mtd1*_D	GATATCATGGGTGATAAATTGGGTA
*tpk2*_A	TACAATTCTGGCCTTCTTACCTAAA
*tpk2*_B	TTGCTACAGGATTAGGAAATCTTTG
*tpk2*_D	TAATTTTTGCACTGAGATCATGAGA
*yap5*_A	GTATTTTAGTTTACCTATTGGGCCG
*yap5*_B	ATCTGTTTCCAGTTCTTTACAAACG
*yap5*_D	TATAGGCATAGTAAGCGGTACCTTG
*yct1*_A	CAATAGTGTTCGAAGTTCTTCCATT
*yct1*_B	GTGAATATTTTCCATAACGAAATGC
*yct1*_D	AGGCTAGAGGGGTTACTAGTCTCTG
*yor387c*_A*	TTCGGATCTTTCCTAAACGG
*yor387c*_C*	ACTCTTGGGCAGACACCG
*yor387c*_D*	TGACAAGAAAACCATCTGCG
*vel1*_A	AAGGTAGTAATTTCATGCATTCTCG
*vel1*_C	ATCTTCACCACCAATATCAACACTT
*vel1*_D	TTACTTTTATCTTGTAGGCAAACCG
*zap1*_A	CTGCGATTTACTCTAGGGATCTTC
*zap1*_B	CTCAGATATGTCTTTCTTTGTCGGT
*zap1*_D	CTTCGGTTACCTAGTTGTCACTCAT
yor387c_S1*[#]	TTCTGATAGATTGTACAATCTCAAGAAATCAAGAACAACAACCATACCATGcgtacgctgcaggtcgac
yor387c_S2*[#]	TAAAAAATATACTTAAAATATGTCTACAGATTATGCAGCTGGAAAAAATCAatcgatgaattcgagctcg

*Primers designed in this project. Other sequences were obtained from Yeast Deletion Project [29].
[#]Common sequences are in lower case.

Supplementary Table S6: Genes associated with iron and zinc homeostasis and stress response pathways selected for gene-knockout experiments.

Gene Name	Log_2 fold-change, AMB treatment	Log_2 fold-change, AMB-LF treatment	Ortholog in fungal pathogens	Human Ortholog	Aft1 or Zap1 Target	Mutant Sensitive to AMB	Putative Druggable Domains	Description
**Genes in enriched pathways**								
AFT1	0.34		cgla	No	No	No	No	Regulation of iron homeostasis
ATG1	1.30	0.43	cneo, calb, cgla, afum	Yes	No	No	Yes	Activation of endocytosis
CCH1		0.31	cneo, calb, cgla, afum	Yes	No	No	Yes	Regulation of transmembrane transport
TPK2	0.43		cneo, calb, cgla, afum	Yes	No	No	Yes	Repression of iron uptake genes
VEL1		-5.51	None	No	Zap1	No	No	Putative cell surface glycoprotein
YAP5	0.31		cgla	No	Aft1	No	Yes	Regulation of iron homeostasis
YCT1	0.66	0.65	afum	Yes	Aft1	No	Yes	Imports cysteine and involved in sulfur assimilation
YOR387C		-4.91	None	No	Aft1, Zap1	No	No	Putative cell surface glycoprotein
ZAP1		-2.22	cneo, calb, cgla	Yes	Aft1	Yes	Yes	Regulation of zinc homeostasis
**Genes with potential as drug targets**								
MET32		-0.47	cneo, calb, cgla, afum	No	Aft1	Yes	Yes	Activates genes involved in sulfur assimilation
MTD1	-0.49	0.39	cneo, calb, cgla, afum	Yes	Zap1	Yes	Yes	Folate biosynthesis pathway

a) Indicates that an orthologue of the *S. cerevisiae* gene was identified in the genome of cneo = *C. neoformans*, calb = *C. albicans*, calb = *C. glabrata* and afum = *A. fumigatus*

- **Supplementary Data S1: Significant differentially expressed genes for the AMB and AMB-LF treatments.**
- **Supplementary Data S2: Significant differentially expressed genes grouped into clusters using Self-Organizing Maps.**
- **Supplementary Data S3: Functional enrichment results for significant up- or down-regulated genes from AMB and AMB-LF treatments.**
- **Supplementary Data S4: Functional enrichment results for each cluster of genes from the Self-Organizing Maps.**

References

1. Twine, N. A., Janitz, C., Wilkins, M. R. & Janitz, M. Sequencing of hippocampal and cerebellar transcriptomes provides new insights into the complexity of gene regulation in the human brain. *Neurosci. Lett.* **541,** 263–268 (2013).
2. Cox, M. P., Peterson, D. A. & Biggs, P. J. SolexaQA: At-a-glance quality assessment of Illumina second-generation sequencing data. *BMC Bioinformatics* **11,** 485 (2010).
3. Engel, S. R. *et al.* The Reference Genome Sequence of *Saccharomyces cerevisiae*: Then and Now. *G3* **4,** 389–398 (2014).
4. Langmead, B. & Salzberg, S. L. Fast gapped-read alignment with Bowtie 2. *Nat Methods* **9,** 357–359 (2012).
5. Kim, D. *et al.* TopHat2: accurate alignment of transcriptomes in the presence of insertions, deletions and gene fusions. *Genome Biol* **14,** R36 (2013).
6. Cherry, J. M. *et al. Saccharomyces* Genome Database: the genomics resource of budding yeast. *Nucleic Acids Res* **40,** D700-5 (2012).
7. Robinson, M. D., McCarthy, D. J. & Smyth, G. K. edgeR: a Bioconductor package for differential expression analysis of digital gene expression data. *Bioinformatics* **26,** 139–140 (2010).
8. Love, M. I., Huber, W. & Anders, S. Moderated estimation of fold change and dispersion for RNA-seq data with DESeq2. *Genome Biol* **15,** 550 (2014).

9. Wehrens, R. & Buydens, L. M. C. Self- and super-organising maps in R: the kohonen package. *J. Stat. Softw.* **21,** (2007).

10. Bindea, G. *et al.* ClueGO: a Cytoscape plug-in to decipher functionally grouped gene ontology and pathway annotation networks. *Bioinformatics* **25,** 1091–1093 (2009).

11. Falcon, S. & Gentleman, R. Using GOstats to test gene lists for GO term association. *Bioinformatics* **23,** 257–258 (2007).

12. Dudoit, S., Pollard, K. S. & van der Laan, M. J. in *Bioinformatics and Computational Biology Solutions Using R and Bioconductor* (eds. Gentleman, R. C., Carey, V. J., Huber, W., Irizarry, R. & Dudoit, S.) 251–272 (Springer - Statistics for Biology and Health Series, 2005).

13. Teixeira, M. C. *et al.* The YEASTRACT database: an upgraded information system for the analysis of gene and genomic transcription regulation in *Saccharomyces cerevisiae*. *Nucleic Acids Res.* **42,** D161-6 (2014).

14. Balakrishnan, R. *et al.* YeastMine-an integrated data warehouse for *Saccharomyces cerevisiae* data as a multipurpose tool-kit. *Database (Oxford).* **2012,** bar062 (2012).

15. Mathelier, A. *et al.* JASPAR 2014: an extensively expanded and updated open-access database of transcription factor binding profiles. *Nucleic Acids Res.* **42,** D142–D147 (2014).

16. Gordân, R. *et al.* Curated collection of yeast transcription factor DNA binding specificity data reveals novel structural and gene regulatory insights. *Genome Biol.* **12,** R125 (2011).

17. Pang, C. N. I., Goel, A., Li, S. S. & Wilkins, M. R. A Multidimensional Matrix for Systems Biology Research and Its Application to Interaction Networks. *J. Proteome Res.* **11,** 5204–5220 (2012).

18. Philpott, C. C. & Protchenko, O. Response to iron deprivation in *Saccharomyces cerevisiae*. *Eukaryot. Cell* **7,** 20–27 (2008).

19. Wu, C.-Y. *et al.* Differential control of Zap1-regulated genes in response to zinc deficiency in *Saccharomyces cerevisiae*. *BMC Genomics* **9,** 370 (2008).

20. Eide, D. J. Homeostatic and adaptive responses to zinc deficiency in *Saccharomyces cerevisiae*. *J. Biol. Chem.* **284,** 18565–18568 (2009).

21. Shannon, P. *et al.* Cytoscape: a software environment for integrated models of biomolecular interaction networks. *Genome Res* **13,** 2498–2504 (2003).

22. Carmona-Gutierrez, D. *et al.* Apoptosis in yeast: triggers, pathways, subroutines. *Cell Death Differ* **17,** 763–773 (2010).

23. Belenky, P., Camacho, D. & Collins, J. J. Fungicidal Drugs Induce a Common Oxidative-Damage Cellular Death Pathway. *Cell Rep.* **3,** 350–358 (2013).

24. Fischer, S. *et al.* Using OrthoMCL to assign proteins to OrthoMCL-DB groups or to cluster proteomes into new ortholog groups. *Curr. Protoc. Bioinformatics* **Chapter 6,** Unit 6 12 1-19 (2011).

25. Camacho, C. *et al.* BLAST+: architecture and applications. *BMC Bioinformatics* **10,** 421 (2009).

26. Lee, A. Y. *et al.* Mapping the cellular response to small molecules using chemogenomic fitness signatures. *Science* **344,** 208–11 (2014).

27. Finn, R. D. *et al.* Pfam: the protein families database. *Nucleic Acids Res* **42,** D222-30 (2014).

28. Law, V. *et al.* DrugBank 4.0: shedding new light on drug metabolism. *Nucleic Acids Res* **42,** D1091-7 (2014).

29. Giaever, G. & Nislow, C. The yeast deletion collection: a decade of functional genomics. *Genetics* **197,** 451–465 (2014).

30. Kelly, D. E., Lamb, D. C. & Kelly, S. L. Genome-wide generation of yeast gene deletion strains. *Comp. Funct. Genomics* **2,** 236–242 (2001).

31. Janke, C. *et al.* A versatile toolbox for PCR-based tagging of yeast genes: new fluorescent proteins, more markers and promoter substitution cassettes. *Yeast* **21,** 947–962 (2004).

32. Gietz, R. D. & Woods, R. A. Transformation of yeast by lithium acetate/single-stranded carrier

DNA/polyethylene glycol method. *Methods Enzym.* **350,** 87–96 (2002).
